# GIT1 protects against breast cancer growth through negative regulation of Notch

**DOI:** 10.1038/s41467-022-28631-y

**Published:** 2022-03-22

**Authors:** Songbai Zhang, Ayako Miyakawa, Malin Wickström, Cecilia Dyberg, Lauri Louhivuori, Manuel Varas-Godoy, Kati Kemppainen, Shigeaki Kanatani, Dagmara Kaczynska, Ivar Dehnisch Ellström, Lotta Elfman, Pauliina Kronqvist, Heli Repo, Katsuhiko Mikoshiba, Cecilia Sahlgren, John Inge Johnsen, Per Uhlén

**Affiliations:** 1 Department of Medical Biochemistry and Biophysics, Karolinska Institutet, Stockholm, Sweden; 2 Department of Molecular Medicine and Surgery, Karolinska University Hospital, Stockholm, Sweden; 3 Department of Women’s and Children’s Health, Karolinska Institutet, Stockholm, Sweden; 4 Centro de Biología Celular y Biomedicina (CEBICEM), Facultad de Medicina y Ciencia, Universidad San Sebastián, Santiago, Chile; 5 Turku Bioscience, Åbo Akademi University and University of Turku, Turku, Finland; 6 Faculty of Science and Engineering, Åbo Akademi University, Turku, Finland; 7 Department of Pathology, University of Turku, Turku, Finland; 8 Shanghai Institute for Advanced Immunochemical Studies, ShanghaiTech University, Shanghai, China; 9 RIKEN Center for Life Science Technologies (CLST), Chuo-ku, Kobe Japan; 10 Department of Biomolecular Science, Faculty of Science, Toho University, Chiba, Japan; 11 Institute for Complex Molecular Systems, Eindhoven University of Technology, Eindhoven, the Netherlands

**Keywords:** Breast cancer, Intracellular signalling peptides and proteins

## Abstract

Hyperactive Notch signalling is frequently observed in breast cancer and correlates with poor prognosis. However, relatively few mutations in the core Notch signalling pathway have been identified in breast cancer, suggesting that as yet unknown mechanisms increase Notch activity. Here we show that increased expression levels of GIT1 correlate with high relapse-free survival in oestrogen receptor-negative (ER(-)) breast cancer patients and that GIT1 mediates negative regulation of Notch. GIT1 knockdown in ER(-) breast tumour cells increased signalling downstream of Notch and activity of aldehyde dehydrogenase, a predictor of poor clinical outcome. GIT1 interacts with the Notch intracellular domain (ICD) and influences signalling by inhibiting the cytoplasm-to-nucleus transport of the Notch ICD. In xenograft experiments, overexpression of GIT1 in ER(-) cells prevented or reduced Notch-driven tumour formation. These results identify GIT1 as a modulator of Notch signalling and a guardian against breast cancer growth.

## Abstract (web summary)

Notch signalling is reported to be hyperactivated in oestrogen receptor-negative (ER-) breast cancer. Here the authors show that G protein-coupled receptor kinase-interacting protein 1 (GIT1) negatively regulates Notch signalling and tumour growth in ER- breast cancer by blocking Notch ICD nuclear translocation.

## Introduction

Breast cancer affects ~10% of women during their lifetime, and thus is a major medical and societal burden^1^. Breast cancer is a heterogeneous disease that can be classified based on the presence or absence of the oestrogen receptor, i.e., ER(+) or ER(−) breast cancers. Hormonal (antioestrogen) therapies are relatively effective for ER(+) tumours; however, fewer treatment options are available for ER(−) breast cancer, particularly for so-called triple-negative breast cancers (TNBCs), which are negative not only for ER but also for the progesterone receptor and HER2 receptor. Identification of molecular mechanisms associated with ER(−) status is therefore warranted, as these mechanisms may represent novel therapeutic targets. Notch signalling has emerged as an interesting candidate^2–4^. The link between hyperactivated Notch signalling and poor prognosis is well established for breast cancer, particularly for ER(−) forms^5–8^. Crosstalk between oestrogen signalling and Notch signalling has been reported^9–11^, and Notch downstream signalling is higher in ER(−) breast cancers than in ER(+) breast cancers, in which oestradiol inhibits Notch signalling^9^. Oestradiol also alters the intracellular distribution of Notch^9^, and ER(−) cells exhibit a more pronounced nuclear distribution of Notch than ER(+) cells^12,13^. Moreover, ER(−) breast cancer cell lines are sensitive to Notch inhibitors^9^, but the nature of the dysregulation of Notch signalling in ER(−) breast cancer has not been fully elucidated.

Notch signalling is an evolutionarily conserved cell–cell communication mechanism in which transmembrane ligands on one cell activate Notch on an adjacent cell^2,14,15^. Receptor–ligand interaction leads to proteolytic cleavage of the receptor by an ADAM metalloprotease (TACE) and γ-secretase. Cleavage by γ-secretase liberates the intracellular domain of Notch (Notch ICD), which migrates to the nucleus, where it regulates Notch target genes and modulates cell fate decisions. The Notch ICD has four nuclear localization signal (NLS1-4) sequences, of which NLS3 and NLS4 are generally recognized as being responsible for the cytoplasmic-nuclear transition of the Notch ICD^16,17^. Although the molecular architecture of the Notch pathway is simple, Notch signalling regulates cell fate decisions in most organs of the body and at different steps during cell lineage progression. The diverse roles of Notch signalling indicate that there are additional control steps in this pathway, which are probably mediated by proteins outside the core signalling pathway. Consistent with this reasoning, surprisingly few mutations exist in genes that encode core Notch pathway components in breast cancer. Numb, a negative regulator of Notch, is frequently lost in breast cancer^6^, but other Notch-regulating proteins remain to be identified.

In this work, we sought to explain why Notch signalling is elevated in ER(−) breast cancer. We identified G protein-coupled receptor kinase-interacting protein 1 (GIT1), an evolutionarily conserved^18,19^ and ubiquitous cytoplasmic adaptor protein involved in multiple cell signalling pathways^20^, as a modulator of Notch signalling in breast cancer and a predictor of poor prognosis in human ER(−) breast cancer. GIT1 interacts with NLS1 and NLS2 of the Notch ICD and negatively regulates Notch signalling by blocking the transition of the Notch ICD from the cytoplasm to the nucleus, a mode of Notch regulation not previously recognized. Increased GIT1 expression abrogated or reduced the development of tumours in mouse xenograft models via downregulation of Notch activity.

## Results

### GIT1 levels are prognostic for ER(−) breast cancer

**Fig. 1:**
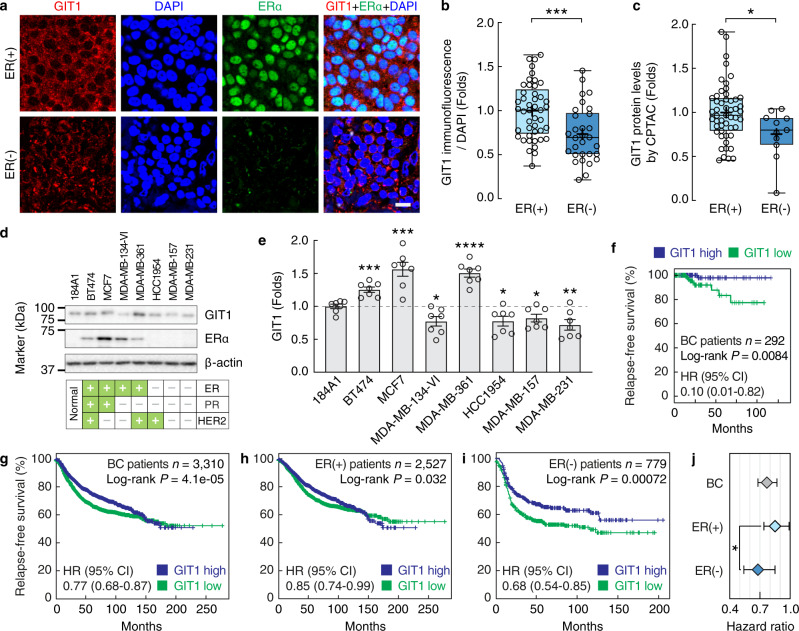
Downregulated GIT1 expression in ER(−) breast tumours is associated with poor relapse-free survival in patients. **a**, **b** Immunostaining of GIT1 and ERα in one ER(+) and one ER(−) patient breast tumour section (**a**) and the quantitative analysis of GIT1 immunofluorescence relative to DAPI (**b**; ER(+) (*n* = 45) versus ER(−) (*n* = 30), *P* = 0.0006, *t* test). Scale bar, 10 μm. **c** Mass spectrometry data from CPTAC of the GIT1 protein levels in ER(+) and ER(−) patients (ER(+) (*n* = 51) versus ER(−) (*n* = 11), *P* = 0.024, *t* test). **d**, **e**, Western blots of GIT1 in various human breast cancer cells (**d**) and the quantitative analysis (**e**; *n* = 7 independent biological replicates, 184A1 versus BT474, *P* = 0.0004; MCF7, *P* = 0.0003; MDA-MB-134-VI, *P* = 0.015; MDA-MB-361, *P* < 0.0001; HCC1954, *P* = 0.017; MDA-MB-157, *P* = 0.023; MDA-MB-231, *P* = 0.0067; *t* tests). ER, oestrogen receptor. PR, progesterone receptor. HER2, human epidermal growth factor receptor 2. **f** Kaplan–Meier plot of relapse-free survival of the breast cancer patients from the TCGA database^59^ stratified by GIT1 expression (*n* = 292, *P* = 0.0084, log-rank test). **g**–**i** Kaplan–Meier plots of relapse-free survival of all breast cancer (BC) patients (**g**; *n* = 3,310, *P* < 0.0001, log-rank test), ER(+) patients (**h**; *n* = 2527, *P* = 0.032, log-rank test), and ER(−) patients (**i**; *n* = 779, *P* = 0.0007, log-rank test) from KM-plotter^61^ stratified by GIT1 expression. Hazard ratios (HRs) and their 95% confidence intervals (95%CI) are indicated. **j**, Forest plot of HRs for survival analysis of all BC, ER(+), and ER(−) patients stratified by GIT1 expression (ER(+) (*n* = 2527) versus ER(−) (*n* = 779), *P* = 0.047, one-sided unpaired *t* test). Error bars show 95%CI. All data are shown as the mean ± s.e.m. For the box plots, the centre line shows the median, the plus sign shows the mean, the upper and lower boundaries of the box show the upper and lower quartiles, and the whiskers show the minimum and maximum values. *P < 0.05, **P < 0.01, ***P < 0.001, ****P < 0.0001 by two-sided unpaired *t* tests. Source data are provided as a Source Data file.

We sought to investigate the protein expression pattern and role of GIT1 in tumour samples from patients with ER(+) and ER(−) breast cancer. Assessment of GIT1 immunoreactivity revealed a significantly lower protein level in samples from ER(−) patients than in those from ER(+) patients (Fig. 1a, b; ER(−), *n* = 30; ER(+), *n* = 45; *P* = 0.0006; *t* test). A proteomic analysis also showed lower protein levels of GIT1 in patients with ER(−) breast cancer compared to patients with ER(+) breast cancer (Fig. 1c). Similarly, in three ER(−) cell lines, the GIT1 protein levels were lower than those in the normal, nontransformed breast epithelial cell line 184A1, whereas three of four ER(+) cell lines exhibited elevated GIT1 protein levels (Fig. 1d, e). To determine whether GIT1 expression could predict prognosis in breast cancer patients, we performed a Kaplan–Meier analysis in multiple databases, which revealed a direct correlation between high GIT1 expression and increased relapse-free survival (Fig. 1f, g). When the data were stratified into breast cancers by different ER statuses, a more pronounced distinction between high and low GIT1 was observed in the ER(−) patients (Fig. 1i, j; *n* = 779, *P* = 0.0007, log-rank test, hazard ratio 0.68, 95% confidence interval (CI) 0.54–0.85) than in the ER(+) patients (Fig. 1h, j; *n* = 2527, *P* = 0.032, log-rank test, hazard ratio 0.85, 95% CI 0.74–0.99). The influence of oestrogen on GIT1 expression was then tested in a series of experiments. We found that 17β-oestradiol increased GIT1 immunoreactivity in ER(+) breast cancer cells, whereas fulvestrant and tamoxifen blocked this effect (Supplementary Fig. 1). Notably, GIT1 was unaffected by 17β-oestradiol in ER(−) breast cancer cells. Taken together, these data demonstrated that GIT1 is expressed at lower protein levels in ER(−) breast tumours than ER(+) tumours and that ER(−) breast cancer patients with high levels of GIT1 have a better prognosis than those with low levels.

### GIT1 negatively regulates Notch signalling and tumour growth

**Fig. 2:**
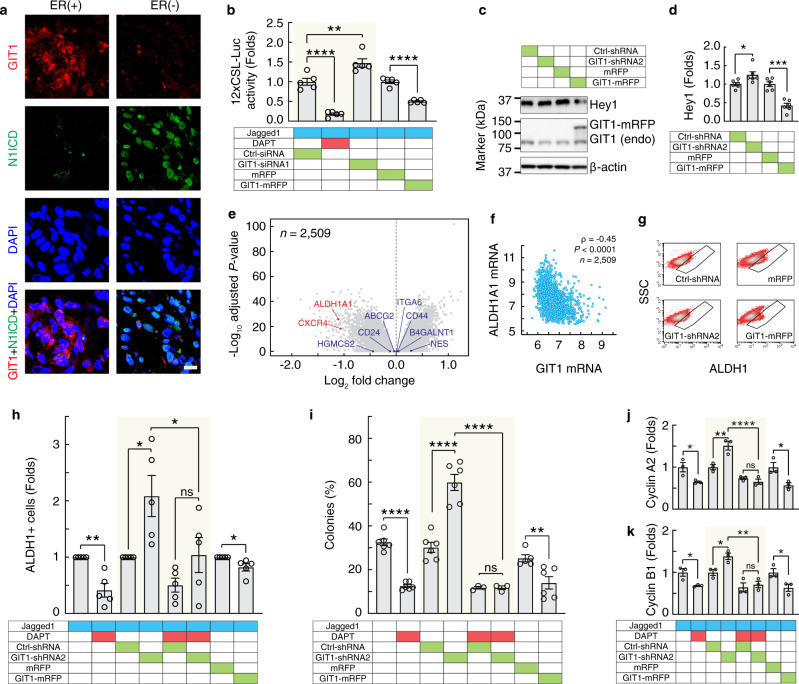
GIT1 regulates Notch signalling, ALDH1 activity, and colony formation. **a** Immunostaining of GIT1 and Notch1 ICD (N1ICD) in one ER(+) and one ER(−) breast cancer sample. Nuclei were detected using DAPI. Images from representative micrographs; the experiment was repeated *n* = 6 times for ER(+) samples and *n* = 4 times for ER(−) samples with similar results. Scale bar, 10 μm. **b**–**d** Luciferase reporter assays of 12xCSL-Luc (**b**; *n* = 5, Ctrl-siRNA versus DAPT, *P* < 0.0001; Ctrl-siRNA versus GIT1-siRNA1, *P* = 0.0038; one-way ANOVA, *F*_2,12_ = 64.17, *P* < 0.0001 (shaded area); mRFP versus GIT1-mRFP, *P* < 0.0001; *t* tests) and western blots of Hey1 (**c**) and the quantitative analysis (**d**; *n* = 6, Ctrl-shRNA versus GIT1-shRNA2, *P* = 0.030; mRFP versus GIT1-mRFP; *P* = 0.0002; *t* tests) in MDA-MB-231 cells treated as indicated. **e**, **f** Volcano plot of differentially expressed genes for high and low GIT1 **e**; *n* = 2509 patients) and a correlation analysis between GIT1 and ALDH1A1 mRNA expression (**f**; *n* = 2509 patients, Spearman *ρ* = −0.45, *P* < 0.0001) in breast cancer samples from the METABRIC database^63^. Breast cancer stemness genes are indicated. **g**, **h** Flow cytometric analysis of Aldefluor-assayed MDA-MB-231 cells treated as indicated (**g**) and the quantitative analysis (**h**; *n* = 5, vehicle versus DAPT, *P* = 0.0014, *t* test; Ctrl-shRNA versus GIT1-shRNA2, *P* = 0.031; GIT1-shRNA2 versus GIT1-shRNA2 + DAPT, *P* = 0.040; Ctrl-shRNA + DAPT versus GIT1-shRNA2 + DAPT, *P* = 0.44; one-way ANOVA, *F*_3,16_ = 7.216, *P* = 0.0028 (shaded area); mRFP versus GIT1-mRFP, *P* = 0.025, *t* test). SSC, side scatter. **i** Clonogenic assay of MDA-MB-231 cells treated as indicated (*n* = 6, vehicle versus DAPT, *P* < 0.0001, *t* test; Ctrl-shRNA versus GIT1-shRNA2, *P* < 0.0001; GIT1-shRNA2 versus GIT1-shRNA2 + DAPT, *P* < 0.0001; Ctrl-shRNA + DAPT (*n* = 3) versus GIT1-shRNA2 + DAPT (*n* = 3), *P* = 1.00; one-way ANOVA, *F*_3,14_ = 57.84, *P* < 0.0001 (shaded area); mRFP (*n* = 5) versus GIT1-mRFP (*n* = 6), *P* = 0.010, *t* test). **j**, **k** Quantitative analyses of Cyclin A (**j**; *n* = 3, vehicle versus DAPT, *P* = 0.036, *t* test; Ctrl-shRNA versus GIT1-shRNA2, *P* = 0.0032; GIT1-shRNA2 versus GIT1-shRNA2 + DAPT, *P* < 0.0001; Ctrl-shRNA + DAPT versus GIT1-shRNA2 + DAPT, *P* = 0.84; one-way ANOVA, *F*_3,8_ = 32.06, *P* < 0.0001 (shaded area); mRFP versus GIT1-mRFP, *P* = 0.028, *t* test) and Cyclin B1 (**k**; *n* = 3, vehicle versus DAPT, *P* = 0.024, *t* test; Ctrl-shRNA versus GIT1-shRNA2, *P* = 0.049; GIT1-shRNA2 versus GIT1-shRNA2 + DAPT, *P* = 0.0022; Ctrl-shRNA + DAPT versus GIT1-shRNA2 + DAPT, *P* = 0.94; one-way ANOVA, *F*_3,8_ = 15.92, *P* = 0.0010 (shaded area); mRFP versus GIT1-mRFP, *P* = 0.043, *t* test) from western blots of MDA-MB-231 cells treated as indicated. All data are shown as the mean ± s.e.m. *n* denotes the number of biologically independent replicates, unless stated otherwise. *P < 0.05, **P < 0.01, ***P < 0.001, ****P < 0.0001, ns, not significant by two-sided unpaired *t* tests or one-way ANOVA with Tukey’s post hoc comparison. Source data are provided as a Source Data file.

Because Notch activity is enhanced in ER(−) breast tumours^9,21^, we explored the possible relationship between GIT1 and Notch. Immunostaining for the Notch1 ICD revealed ample nuclear staining in the ER(−) patient specimens and, conversely, low immunoreactivity in the ER(+) specimens (Fig. 2a). We next analysed the effects of high and low GIT1 levels in MDA-MB-231 cells, a TNBC cell line that endogenously expresses Notch receptors and ligands^22,23^. Silencing GIT1 expression with both siRNA and shRNA (Supplementary Fig. 2) mimicked the expression difference observed in ER(−) patient samples (Fig. 1a–c) and resulted in an increase in Notch reporter (12xCSL-Luc) expression (Fig. 2b, *n* = 5, *P* = 0.0038, *t* test), whereas overexpression of GIT1 (GIT1-mRFP) reduced the activity of 12xCSL-Luc (*n* = 5, *P* < 0.0001, *t* test), both with or without enhanced Notch signalling by immobilizing Jagged1 ligands^24^ (Supplementary Fig. 3). As a control, treatment with the γ-secretase inhibitor N-[N-(3,5-difluorophenacetyl)-L-alanyl]-S-phenylglycine t-butyl ester (DAPT), which blocks Notch cleavage, inhibited the increase in Notch reporter activity caused by GIT1 knockdown. Consistent with the Notch reporter data, the expression of a protein downstream of Notch, Hey1, was increased upon knockdown of GIT1 with shRNA and was decreased upon GIT1 overexpression (Fig. 2c, d and Supplementary Fig. 3).

To explore GIT1’s ability to influence tumour development and growth, we searched for genes associated with cell tumour stemness in breast cancer^25^. Differential gene expression analysis between breast cancer patients (*n* = 2509) with high and low GIT1 levels revealed significantly increased expression of aldehyde dehydrogenase 1 (ALDH1) and C-X-C chemokine receptor type 4 (CXCR4) in the patients with low GIT1 (Fig. 2e). Because transcriptional activation of CXCR4 in MDA-MB-231 cells is regulated upstream by ALDH1^26^, we focused on the relationship between ALDH1 and GIT1. Intriguingly, we found a moderate but clear negative correlation between ALDH1 and GIT1 in breast cancer patients (Fig. 2f, *n* = 2509, Spearman’s *ρ* = −0.45, *P* < 0.0001). When GIT1 expression was silenced in TNBC cells, we observed a significant increase in the number of ALDH1+ cells, while GIT1 overexpression reversed this effect (Fig. 2g, h and Supplementary Fig. 4, 5; *n* = 5, vehicle versus DAPT, *P* = 0.0014, *t* test; Ctrl-shRNA versus GIT1-shRNA2, one-way ANOVA, *F*_3,16_ = 7.216, *P* = 0.0028 (shaded area); mRFP versus GIT1-mRFP, *P* = 0.025, *t* test). Treatment with DAPT inhibited GIT1’s ability to regulate the population size of ALDH1+ cells.

The colony-forming capacity is an intrinsic cell property that is strongly associated with cancer stemness^27^. We found that knockdown of GIT1 in TNBC cells significantly increased the number of colonies, whereas GIT1 overexpression reduced the number of colonies compared to that of the control cells (Fig. 2i and Supplementary Fig. 6; *n* = 6, vehicle versus DAPT, *P* < 0.0001, *t* test; Ctrl-shRNA versus GIT1-shRNA2, one-way ANOVA, *F*_3,14_ = 57.84, *P* < 0.0001 (shaded area); mRFP versus GIT1-mRFP, *P* = 0.010, *t* test). DAPT abolished GIT1’s ability to enhance or suppress colony formation of TNBC cells. We further analysed the influence of the Notch-GIT1 axis on tumour development by a limiting dilution assay. As expected, GIT1 could not control spheroid formation when only one cell was seeded, likely because Notch signalling is diminished in solitary cells (Supplementary Fig. 6). However, when we seeded two or three cells, significantly more or fewer spheroids were observed in the GIT1 knockdown or overexpression cells, respectively, compared to the controls. After we seeded five cells or more, the stimulatory effect of GIT1 knockdown was saturated, as virtually all (89.9%) control cells generated spheroids; nevertheless, significantly fewer spheroids were observed in the GIT1-overexpressing cells.

Notch signalling regulates the expression levels of Cyclin A2 and B1^9^, which are negative prognostic markers of breast cancer. Overexpression of GIT1 downregulated Cyclin A2 and B1 levels, while knockdown of GIT1 led to increased Cyclin A2 and B1 expression, which was abrogated by DAPT (Fig. 2j, k and Supplementary Fig. 7). The influence of GIT1 on proliferation was also examined by assaying the incorporation of 5-ethynyl-2′-deoxyuridine (EdU) in MDA-MB-231 cells. We detected a significant increase or decrease in EdU-positive cells in the GIT1 knockdown or overexpression cells (Supplementary Fig. 8), respectively. The increase in EdU-positive cells caused by GIT1 knockdown was abrogated by DAPT. Collectively, these data indicate that GIT1 can influence Notch signalling in TNBC cells.

### GIT1 binds directly to the Notch ICD

**Fig. 3:**
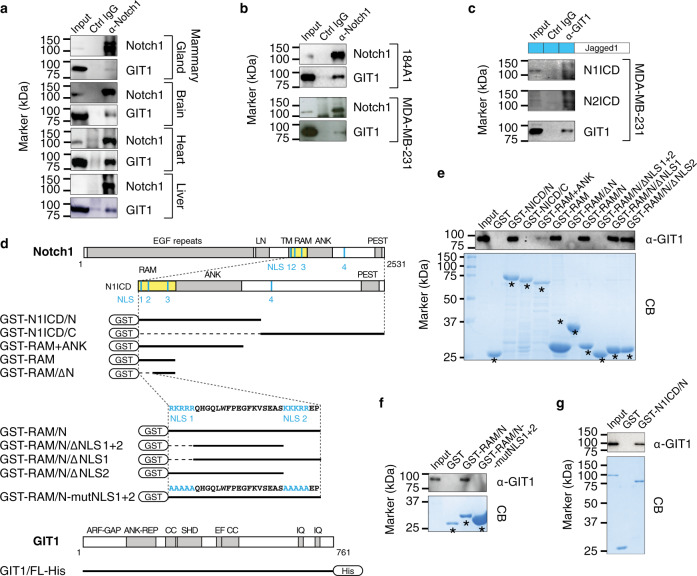
GIT1 directly binds to the Notch intracellular domain. **a**, **b** Co-IP of GIT1 with Notch1 from lysates of the indicated mouse organs (**a**) and human breast cell lines (**b**). **c** Co-IP of the Notch1-2 ICDs with GIT1 from lysates of the MDA-MB-231 cells treated as indicated. **d**, Schematic diagrams of recombinant fusion protein fragments. **e**, **f** GIT1 pulled down by GST Notch fragments from MDA-MB-231 cell lysates. **g** Pulldown of purified GIT1-His by purified GST-N1ICD/N. CB, Coomassie Blue. Images from representative blots; the experiment was repeated *n* = 3 times with similar results. *, indicates recombinant GST-fused peptides. Source data are provided as a Source Data file.

The GIT1-Notch axis established above prompted us to explore the relationship between GIT1 and Notch in more detail. Coimmunoprecipitation (co-IP) experiments revealed an interaction between endogenous GIT1 and Notch1 in mouse mammary glands and other organs (Fig. 3a), as well as in 184A1 and MDA-MB-231 cells (Fig. 3b). GIT2 was not coprecipitated with Notch1 (Supplementary Fig. 9). To determine whether GIT1 binds γ-secretase-cleaved Notch ICDs, we performed co-IP experiments in Jagged1-activated TNBC cells (Fig. 3c), which revealed that GIT1 interacts with the Notch1-2 ICDs. In the DAPT-treated cells, Notch ICDs were not present to coprecipitate with GIT1 (Supplementary Fig. 9).

To more precisely identify the part of the Notch ICD required for this interaction, we generated several glutathione S-transferase (GST)-tagged Notch1 ICD fragments (Fig. 3d) and probed their interaction with GIT1. GIT1 bound the N-terminal portion of the Notch1 ICD encompassing the RAM and ANK domains and the first three NLS sequences (Fig. 3e). The Notch1 ICD RAM domain was sufficient for binding, but removal of the region containing both NLS1 and NLS2 eliminated the GIT1 interaction. Further mutational analysis confirmed that simultaneously mutating both NLS1 and NLS2 abolished the GIT1 interaction (Fig. 3f). To determine whether this interaction is direct or indirect, we purified recombinant His-tagged GIT1 and the GST-tagged N-terminal fragment of the Notch1 ICD and performed a pulldown assay. The purified N-terminal fragment of the Notch1 ICD was bound to the purified GIT1 (Fig. 3g). These data demonstrated that GIT1 directly binds the N-terminal part of the Notch ICD and that the two N-terminal NLS domains are required for binding.

### GIT1 regulates the nuclear translocation of the Notch ICD

**Fig. 4:**
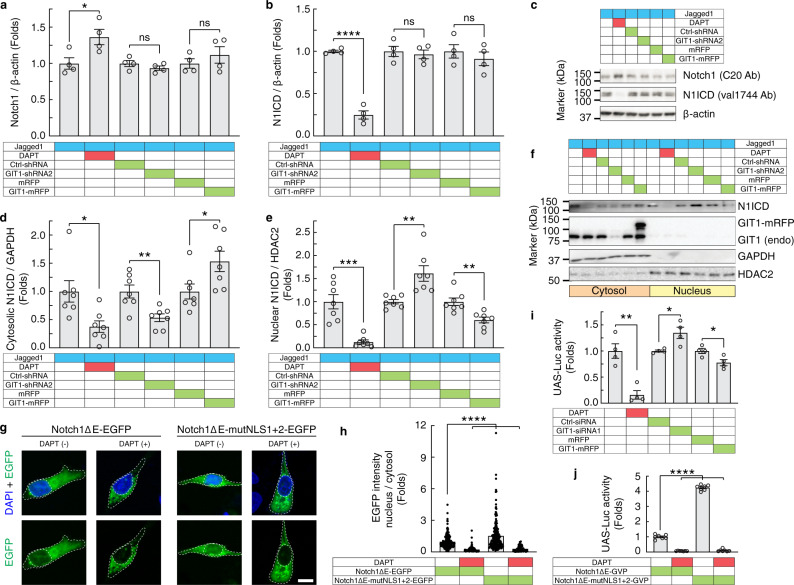
GIT1 regulates the subcellular distribution of the Notch ICD. **a**–**c** Quantitative analyses of Notch1 (**a**; *n* = 4, vehicle versus DAPT, *P* = 0.032; Ctrl-shRNA versus GIT1-shRNA2, *P* = 0.26; mRFP versus GIT1-mRFP, *P* = 0.40; *t* tests) and the Notch1 ICD (N1ICD) (**b**; *n* = 4, vehicle versus DAPT, *P* < 0.0001; Ctrl-shRNA versus GIT1-shRNA2, *P* = 0.67; mRFP versus GIT1-mRFP, *P* = 0.46; *t* tests) western blots (**c**) from lysates of MDA-MB-231 cells treated as indicated. **d**–**f** Quantitative analyses of cytosolic (**d**; *n* = 7, vehicle versus DAPT, *P* = 0.013; Ctrl-shRNA versus GIT1-shRNA2, *P* = 0.0045; mRFP versus GIT1-mRFP, *P* = 0.035; *t* tests) and nuclear (**e**; *n* = 7, vehicle versus DAPT, *P* = 0.0001; Ctrl-shRNA versus GIT1-shRNA2, *P* = 0.0042; mRFP versus GIT1-mRFP, *P* = 0.0025; *t* tests) subcellular fractionation assays with western blots (**f**) from MDA-MB-231 cells treated as indicated. **g**, **h** Confocal images (**g**) and quantitative analysis of EGFP intensities in the cytoplasm and nucleus (**h**; Notch1ΔE−EGFP (*n* = 150 cells) versus: Notch1ΔE−EGFP + DAPT (*n* = 79 cells), *P* < 0.0001; Notch1ΔE-mutNLS1+2−EGFP (*n* = 180 cells), *P* < 0.0001; Notch1ΔE-mutNLS1+2−EGFP + DAPT (*n* = 63 cells), *P* < 0.0001; one-way ANOVA, *F*_3,468_ = 42.82, *P* < 0.0001) of MDA-MB-231 cells treated as indicated. Nuclei were detected using DAPI. **i**, **j** Luciferase reporter assays of Notch1 ICD (UAS-Luc) in MDA-MB-231 cells with knockdown or overexpression of GIT1 (**i**; *n* = 4, vehicle versus DAPT, *P* = 0.0019; Ctrl-siRNA versus GIT1-siRNA1, *P* = 0.020; mRFP versus GIT1-mRFP, *P* = 0.027; *t* tests) or mutated NLS1 and NLS2 (**j**; *n* = 7, Notch1ΔE-GVP versus: Notch1ΔE-GVP + DAPT, *P* < 0.0001; Notch1ΔE-mutNLS1+2-GVP, *P* < 0.0001; Notch1ΔE-mutNLS1+2-GVP + DAPT, *P* < 0.0001; one-way ANOVA, *F*_3,24_ = 1828, *P* < 0.0001). Scale bar, 10 μm. All data are shown as the mean ± s.e.m. *n* denotes the number of biologically independent replicates, unless stated otherwise. *P < 0.05, **P < 0.01, ***P < 0.001, ****P < 0.0001, ns, not significant by two-sided unpaired *t* tests or one-way ANOVA with Tukey’s post hoc comparison. Source data are provided as a Source Data file.

Notch signalling can be modulated at different steps in the signalling cascade^2,14^, and we first examined whether GIT1 influences Notch expression. High and low GIT1 levels had no apparent effect on the combined amount of the Notch1 NEXT (processed by TACE but not by the γ-secretase complex) and the Notch1 ICD (processed by the γ-secretase complex), which generate protein fragments of approximately the same size on a western blot (Fig. 4a, c). Immunoblotting specifically for the Notch1 ICD using an antibody that recognizes only the γ-secretase-cleaved Notch1 ICD (val1744) revealed that there was no change in the level of the Notch1 ICD after GIT1 modulation, whereas DAPT treatment, as expected, produced a strong decrease in the Notch1 ICD level (Fig. 4b, c). The lack of an effect of GIT1 on the Notch1 ICD levels indicates that GIT1 does not affect S3 cleavage of Notch. The synthesis and degradation of Notch1 were also unaffected by modifying the expression level of GIT1 (Supplementary Fig. 10).

In contrast, the intracellular distribution of the Notch1 ICD was altered by GIT1. Knockdown of GIT1 increased the amount of the Notch1 ICD in the nuclear fraction and was accompanied by a corresponding decrease in the cytosolic fraction (Fig. 4d, f; *n* = 7, Ctrl-shRNA versus GIT1-shRNA2, *P* = 0.0045; mRFP versus GIT1-mRFP, *P* = 0.035; *t* tests). Conversely, the overexpression of GIT1 resulted in a decrease in the nuclear Notch1 ICD level, with a concomitant increase in the cytosolic fraction (Fig. 4e, f; *n* = 7, Ctrl-shRNA versus GIT1-shRNA2, *P* = 0.0042; mRFP versus GIT1-mRFP, *P* = 0.0025; *t* tests). Next, we analysed the intracellular distribution of the control Notch1 ICD and the Notch1 ICD with mutated NLS1 and NLS2 generated by endogenous γ-secretase cleavage from the corresponding membrane-tethered forms: Notch1ΔE-EFGP and Notch1ΔE-mutNLS1+2−EGFP (Supplementary Fig. 11). We first confirmed that Notch1ΔE-mutNLS1+2−EGFP could be cleaved by γ-secretase and that the mutated protein could not bind to GIT1. Notch1ΔE-mutNLS1+2−EGFP exhibited stronger nuclear localization than control Notch1ΔE−EGFP (Fig. 4g, h), and this effect was not suppressed by GIT1 overexpression (Supplementary Fig. 12). Moreover, Notch1ΔE-mutNLS1+2+3+4−EGFP had a similar effect on nuclear localization as Notch1ΔE-mutNLS3+4−EGFP. To investigate the cytoplasmic-nuclear distribution in an unbiased fashion, we generated the same NLS mutations in a Notch1ΔE construct containing a GAL4VP16 domain with a UAS-Luc reporter as a readout^28^. Notch1ΔE-GVP exhibited stronger UAS-Luc reporter activity upon GIT1 knockdown in MDA-MB-231 cells and, conversely, less activity upon GIT1 overexpression (Fig. 4i). Mutating both NLS1 and NLS2 in Notch1ΔE-GVP enhanced the UAS-Luc reporter activity (Fig. 4j). The increase in UAS-Luc activity with NLS mutations was unaffected by GIT1 knockdown or overexpression (Supplementary Fig. 12). Together, these experiments suggest that GIT1 anchors the Notch ICD in the cytoplasm, thereby hindering its nuclear translocation.

### High GIT1 levels protect against xenograft tumour growth by blocking Notch signalling

**Fig. 5:**
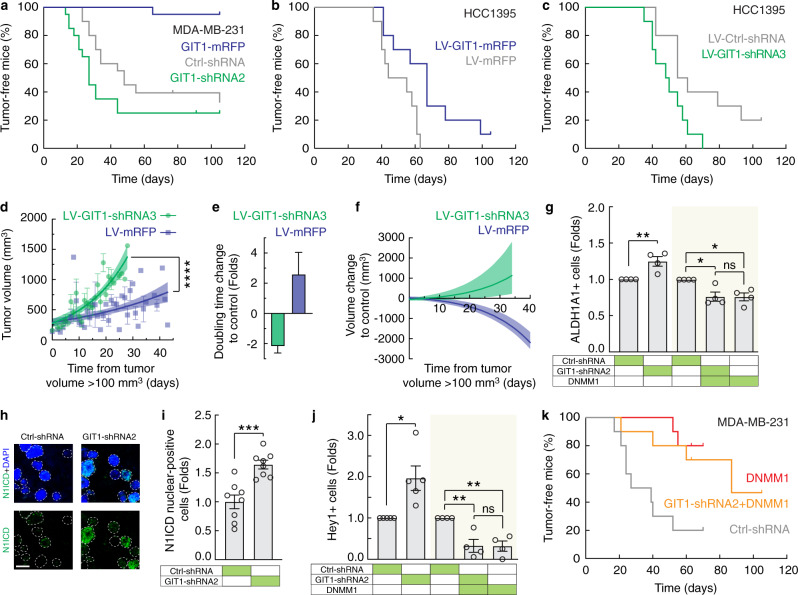
GIT1 suppresses the in vivo growth of TNBC cells. **a**–**c**, **k** Kaplan–Meier plots showing the percentage of tumour-free mice out of ten mice transplanted with MDA-MB-231 (**a**, **k**) or HCC1395 cells (**b**, **c**) stably expressing the indicated plasmids. All injection sites were assessed independently, and a tumour was defined as >100 mm^3^ (MDA-MB-231) and >500 mm^3^ (HCC1395). Comparisons of Kaplan–Meier curves, MDA-MB-231 implants: Ctrl-shRNA versus GIT1-mRFP, *P* < 0.0001; Ctrl-shRNA versus GIT1-shRNA2, *P* = 0.12 (**a**); HCC1395 implants: LV-mRFP versus LV-GIT1-mRFP, *P* = 0.012 (**b**); LV-Ctrl-shRNA versus LV-GIT1-shRNA3, *P* = 0.025 (**c**); MDA-MB-231 implants: Ctrl-shRNA versus DNMM1, *P* = 0.0038; Ctrl-shRNA versus GIT1-shRNA2 + DNMM1, *P* = 0.030; DNMM1 versus GIT1-shRNA2 + DNMM1, *P* = 0.58 (**k**); log-rank tests. **d** Scatter plot of tumour volume over time in the mice transplanted with HCC1395 cells expressing LV-GIT1-shRNA3 and LV-GIT1-mRFP. Time is days from the tumour volume >100 mm^3^. Solid line, exponential regression. Shaded area, 95% confidence bands. Comparison of growth rate constants *k* (Methods) for LV-GIT1-shRNA3 (*n* = 290 tumour measurements, *k* = 0.057) and LV-GIT1-mRFP (*n* = 440 tumour measurements, *k* = 0.023), *P* < 0.0001; *F*-test. **e**, **f** Tumour doubling time (**e**) and volume change (**f**) for LV-GIT1-shRNA3 versus LV-Ctrl-shRNA and LV-GIT1-mRFP versus LV-mRFP. Solid line, exponential regression. Shaded area, 95% confidence interval. **g**, **j** Flow cytometric analysis of ALDH1A1+ cells (**g**; *n* = 4, Ctrl-shRNA versus GIT1-shRNA2, *P* = 0.0090, *t* test; Ctrl-shRNA versus GIT1-shRNA2, *P* = 0.019; Ctrl-shRNA versus DNMM1, *P* = 0.018; GIT1-shRNA2 + DNMM1 versus DNMM1, *P* = 1.00; one-way ANOVA, *F*_2,9_ = 7.877, *P* = 0.011 (shaded area)) and Hey1+ cells (**j**, *n* = 4, Ctrl-shRNA versus GIT1-shRNA2, *P* = 0.012, *t* test; Ctrl-shRNA versus GIT1-shRNA2, *P* = 0.0063; Ctrl-shRNA versus DNMM1, *P* = 0.0059; GIT1-shRNA2 + DNMM1 versus DNMM1, *P* = 1.00; one-way ANOVA, *F*_2,9_ = 11.61, *P* = 0.0032 (shaded area)) in MDA-MB-231 xenograft tumours. **h**, **i** Confocal images of the Notch1 ICD (N1ICD)-immunolabelled MDA-MB-231 xenograft tumours (**h**) and quantitative analysis (**i**; *n* = 8 tumours, *P* = 0.0004, *t* test). Nuclei were detected using DAPI. Scale bar, 5 μm. All data are shown as the mean ± s.e.m. *n* denotes the number of biologically independent replicates, unless stated otherwise. *P < 0.05, **P < 0.01, ***P < 0.001, ****P < 0.0001, ns, not significant by two-sided unpaired *t* test, *F*-test, or one-way ANOVA with Tukey’s post hoc comparison. Source data are provided as a Source Data file.

To determine whether the GIT1-Notch axis plays a role in tumour progression, we assessed the growth of genetically modified MDA-MB-231 and HCC1395 cells xenografted into immunodeficient nude mice. Excitingly, GIT1 overexpression substantially reduced tumour formation in these animals (Fig. 5a, b; MDA-MB-231 implants, Ctrl-shRNA versus GIT1-mRFP, *P* < 0.0001, log-rank test). Among twenty MDA-MB-231 implants, only one GIT1-overexpression tumour was detected, which required 63 days for initiation and 105 days to achieve a size of 0.1 mm^3^. Conversely, knockdown of GIT1 expression by two different shRNAs accelerated tumour formation and growth in the xenografted animals (Fig. 5a, c; MDA-MB-231 xenograft tumours, Ctrl-shRNA (*n* = 13) versus GIT1-shRNA2 (*n* = 15), *P* = 0.12, log-rank test, *P* = 0.0063, Mann–Whitney *U*-test). Growth curves also showed that GIT1 knockdown and overexpression had a profound effect on tumour growth rates in vivo (Fig. 5d–f; doubling time: 2.60-fold increase for GIT1-mRFP, 2.16-fold decrease for GIT1-shRNA3). Consistent with these findings, the number of ALDH1+ cells in the GIT1 knockdown tumours was significantly higher than that in the control tumours (Fig. 5g). Immunostaining and flow cytometry revealed that the nuclear Notch1 ICD and Hey1 levels, respectively, were enhanced in the GIT1 knockdown tumours compared to the control tumours (Fig. 5h–j). The number of EdU-positive cells in the GIT1 knockdown tumours was significantly higher than in the controls (Supplementary Fig. 13). To further assess whether the increased tumour growth observed in the GIT1 knockdown tumours was caused by an increase in Notch signalling, we used a dominant-negative mutant of Mastermind-like 1 (DNMM1), which inhibits the interaction between the Notch ICD and CSL and thus acts as a negative regulator of Notch signalling^29,30^. In xenografted mice, stable expression of DNMM1 alone prevented tumour development, and expression of DNMM1 together with GIT1 shRNA dramatically attenuated the tumour growth-promoting effect of GIT1 knockdown (Fig. 5k). Moreover, DNMM1 significantly reduced the number of ALDH1+ and Hey1+ cells in the xenografted tumours (Fig. 5g, j). In summary, these data indicated that high levels of GIT1 protect against the initiation of tumour growth through attenuation of Notch signalling and that loss of GIT1 leads to accelerated tumour formation via elevated Notch signalling.

## Discussion

It is increasingly apparent that dysregulated Notch signalling is associated with various types of cancer^31,32^, including ER(−) breast cancer. However, because few mutations in the core Notch pathway have been reported in this type of cancer^33^, proteins outside the core Notch pathway likely influence the Notch activation state^2^. In this study, we identified GIT1 as a Notch-interacting protein in breast tumour cells. GIT1 directly interacts with the Notch ICD and regulates the nuclear entry of the Notch ICD in ER(−) breast tumour cells. Knockdown of GIT1 in ER(−) breast tumour cells elevates Notch activity and increases ALDH1+ cell count. GIT1 also plays a key role in controlling breast tumour growth in vitro and in vivo through its regulation of Notch signalling. Additionally, GIT1 could serve as a biomarker in ER(−) breast cancer.

The level of GIT1 had a marked effect on tumour xenograft formation by TNBC cells. In MDA-MB-231 cells with elevated GIT1 levels, tumour formation was nearly abolished, and only one small tumour was recorded after 105 days. This effect is likely mediated by GIT1 controlling Notch signalling because knockdown of GIT1 increased Notch activities and tumour growth, whereas inhibition of Notch abrogated the increase in tumour growth. However, the function of GIT1 in tumour biology is complex, and involves multiple signalling pathways. In lung cancer, GIT1 stimulated cancer cell mobility and metastasis by altering the activity of Rac/Cdc42^34^. GIT1 acts as a subunit of a larger protein scaffold by binding to the Rac/Cdc42 guanine nucleotide exchange factors ARHGEF6/7 (known as PIXs) and interacting with multiple proteins, including members of the p21 protein (Cdc42/Rac)-activated kinase (PAK) family and focal adhesion kinase (FAK) (reviewed in ref. ^20^). PIX-GIT1 also modulates focal adhesion formation, invasion, and metastasis of oral squamous cell carcinoma via regulation of FAK, paxillin, ERK1/2, and MMP2/9^35^. Furthermore, the growth of liver and colon cancer is regulated by a signal cascade in which GIT1 activates ERK plus MEK by interacting with MAT2B^36^. Intriguingly, all these cancer types have been associated with Notch signalling^15,37^. A study using animals injected via the tail vein with sublines of highly metastatic breast cancer cells reported that inhibition of GIT1 expression reduced cell migration/invasion and lymph node metastasis through FAK and paxillin signalling^38^. Our animal studies with xenografted tumours showed that knockdown of GIT1 expression accelerated the development and growth of the primary tumour. Thus, GIT1 appears to have distinct functions in the growth and migration of breast cancer cells. During development, GIT1 acts as a scaffold for several protein partners, forming complexes that modulate critical signalling pathways such as the Hippo-Yap, calcium, AKT-mTOR, and epidermal growth factor receptor pathways^20,39–41^. Deficiency of GIT1 in bone marrow mesenchymal stem cells and stalk cells has been reported to impair angiogenesis by signalling cascades in which GIT1 interacts with the NF-κB essential modulator^42^ and RPB-J^43^, respectively, modulating Notch activity. In breast cancer, our data revealed that GIT1 directly interacts with the Notch ICD in the cytoplasm, thus preventing nuclear translocation and transcription of Notch downstream target genes.

The Notch signalling pathway has a simple molecular design but functions in numerous biological contexts^44,45^ and is differentially and crucially affected by low or high dose Notch activation^46,47^, suggesting that multiple mechanisms fine-tune the signalling output. Numb is an important regulator of Notch in breast cancer, and Numb loss-of-function via deletion or downregulation is observed in ∼50% of breast tumours^6,8^. Interestingly, our results showed similarities between GIT1 and Numb in its negative regulation of Notch. Moreover, monoubiquitylation of Notch at the plasma membrane regulates receptor internalization^48^, and atypical protein kinase C phosphorylates membrane-tethered Notch and controls the transition from late endosomes to the nucleus of the processed Notch^49^. GIT1 appears to control the cytoplasmic-nuclear transition by binding to NLS1 and NLS2 in the Notch ICD. There are four putative NLSs in the Notch ICD (Fig. 3d and Supplementary Fig. 11). In general, only NLS3 and NLS4 are considered responsible for the nuclear translocation of the Notch ICD^16,17^. Here, we determined that NLS1 and NLS2 are also involved in the cytoplasmic-nuclear transition of the Notch ICD. In contrast to previous reports showing that deletion or mutation of NLS3 and/or NLS4 impairs nuclear entry of the Notch ICD, we determined that the mutation of both NLS1 and NLS2, which abrogate the GIT1 interaction, enhanced the nuclear localization of the Notch ICD. Whether GIT1 binding to these two NLSs inhibits nuclear transport via importin-α-mediated nuclear translocation of the Notch ICD^16^ remains to be established. Notably, Pim kinase can phosphorylate the Notch1 ICD, leading to enhanced nuclear entry and activation^50^, and GIT1 might block access of Pim kinase when bound to the Notch1 ICD. Overall, GIT1 adds to an emerging view of an elaborate control mechanism of Notch signalling in distinct steps from the membrane-tethered receptor at the plasma membrane to activated Notch ICD in the nucleus.

From a breast cancer diagnostics perspective, it is interesting to note that the GIT1 protein levels were lower in ER(−) than in ER(+) breast cancer and that lower GIT1 levels in ER(−) breast cancer patients correlated with poor relapse-free survival. Previous work has shown that ER(−) breast cancer cells exhibit higher nuclear accumulation of the Notch ICD and stronger Notch transcriptional activity than ER(+) breast cancer cells^9,21^. Our data provide a reasonable explanation for the observed higher Notch activity in ER(−) breast tumours: the lower protein levels of GIT1 in ER(−) breast tumours not only serve as a prognostic biomarker but also lead to elevated Notch nuclear localization and activity. Others have reported that enriched populations of ALDH1+ cells correlate with poor clinical outcomes^25,51,52^, suggesting the use of ALDH1 as a prognostic biomarker for breast cancer patients. Interestingly, we discovered that silencing GIT1 in ER(−) breast cancer cells resulted in an expanded ALDH1+ population and enhanced clonogenic capacity, indicating that GIT1 is a critical negative modulator of ALDH1. Inhibiting Notch diminished GIT1’s ability to regulate ALDH1. These results are consistent with previous observations that Notch signalling can activate ALDH1A1 to promote breast cancer stem cells^53^. Moreover, in ER(+) cells, ER antagonists have been shown to increase breast cancer stem cell activity through Notch activation^10^. Another study in ER(+) cells showed that oestradiol reduces Notch activity and Notch ICD nuclear distribution, while ER inhibitors block this effect^9^. Our data demonstrated that oestradiol elevates GIT1 protein levels in ER(+) cells, which may indicate that the effect of oestradiol levels on Notch is indirect and occurs via modulation of GIT1 expression.

In conclusion, the discovery of the GIT1-Notch axis in ER(−) breast cancer sheds light on the control of Notch signalling and identifies GIT1 as a guardian against breast cancer growth.

## Methods

### Cell cultures and stable cell lines

The cell lines 184A1, BT474, HCC1395, HCC1954, MCF7, MDA-MB-134-VI, MDA-MB-157, MDA-MB-231, and MDA-MB-361 were purchased from ATCC (American Type Culture Collection, Manassas, VA, USA) and were certified mycoplasma free. The cell lines MDA-MB-231^21^ and 184A1^54^ were cultured as previously described. BT474, HCC1395, and HCC1954 cells were maintained in RPMI 1640 medium (Cat. 31870-025, Invitrogen, Waltham, MA, USA) supplemented with 10% foetal bovine serum (FBS, Cat. 25149-079, Invitrogen). MDA-MB-231, MDA-MB-134-VI, MCF7, MDA-MB-361, and MDA-MB-157 cells were maintained in Dulbecco’s modified Eagle’s medium (DMEM, Cat. 41965, Invitrogen) supplemented with 10% FBS. All cell culture media were supplied with 1% penicillin-streptomycin (Cat. 15140122, Invitrogen) or antibiotic-antimycotic (Cat. 15240062, Invitrogen). Stably transfected cell lines were selected and maintained using Zeocin (200 μg/mL, Cat. R25001, Invitrogen) for transfection of GIT1-mRFP and mRFP, while Puromycin (0.5 μg/mL, Cat. A11138-03, Invitrogen) was used for the transfection of Ctrl-shRNA and GIT1-shRNA2 and infection of LV-Ctrl-shRNA and LV-GIT1-shRNA3-4, respectively. Positive colonies were picked, expanded, and validated by western blotting and quantitative-PCR (Q-PCR). For all experiments, the GIT1 expression was confirmed before use and for xenograft experiments just before injection into animals (Fig. 2c and Supplementary Fig. 2).

### Breast cancer patient material

All breast cancer patient material was precharacterized by immunohistochemical staining for ERα, PR, and Her2, as part of the clinical diagnostic routines of the Department of Pathology, Turku University Hospital, Turku, Finland. Briefly, ERα, PR, and Her2 immunostainings was carried out using the fully automated immunostaining machine BenchMark XT (Roche Diagnostics/Ventana Medical Systems, Tucson, AZ, USA). Antigen retrieval and incubation times with ready-to-use antibodies were optimized for the UltraView Universal DAB Detection Kit. Positive controls were adopted from the immunohistochemical routine procedure at the department. Interpretations of the ERα immunostaining were performed according to generally accepted international guidelines at the time of the study with a 1% threshold chosen for allocating patients into negative and positive expression subgroups^55^. Experiments using human samples were ethically approved by the Ethical Committee of Turku University Hospital in Finland (Ethical number: 6/2002), and subjects were informed that their participation was voluntary.

### Notch activation and inhibition

Activation of Notch signalling by immobilized ligands was performed, with modification, as previously described^24^. Briefly, 6-well-plates were coated with 50 μg/ml Protein G (Cat. 10–1201, Invitrogen) in PBS overnight at room temperature (RT). The coated plates were washed three times with PBS and then blocked with 1% BSA in PBS at RT for 2 h. The blocked plates were washed three times with PBS and incubated with 2 μg/ml recombinant Jagged1-FC chimera (Cat. 599-JG, R&D Systems, Minneapolis, MN, USA) in 1% BSA/PBS at RT for 2–4 h. After three washes with PBS, the cells were immediately seeded on the coated plates for one day. For inhibition of Notch signalling, N-[N-(3,5-difluorophenacetyl-L-alanyl)]-S-phenylglycine tert-butyl ester (DAPT, Cat. D5942, Sigma-Aldrich, St. Louis, MO, USA) was solubilized in DMSO vehicle at 25 mM for a stock solution and applied to the cell culture medium at 50 μM.

### Cell lysate extracts and western blotting

Total cell lysates were extracted using lysis buffer (10 mM HEPES (PH 7.4), 100 mM NaCl, 1 mM EDTA, 1 mM 2ME, 0.1% Triton X-100) with protease inhibitor cocktail tablets (Cat. 11836170001, Roche Diagnostics GmbH, Basel, Switzerland), then sonicated, and cleared by centrifugation at 14,000 g for 10 min. The relative protein concentration was determined using a Nanodrop 2000 system (Thermo Fisher Scientific, Waltham, MA, USA). Samples were subjected to SDS-PAGE, and proteins were transferred onto nitrocellulose membranes. Western blotting was performed as previously described^39^. Subcellular protein fractionation was performed using a Subcellular Fractionation Kit (Cat. 78840, Thermo Fisher Scientific), following the manufacturer’s protocol. The following antibodies were used (WB, Western Blotting; IP, Immunoprecipitation): anti-GIT1 (WB 1:4000, IP 6 μg, Cat. N39B/8, NeuroMab), anti-GIT1 (H-170) (WB 1:100, Cat. sc-13961, Santa Cruz Biotechnology), anti-GIT2 (WB 1:50, Cat. N83/48, NeuroMab, Davis, CA, USA), anti-ERα (HC20) (WB 1:1000, Cat. sc-543, Santa Cruz Biotechnology, Dallas, TX, USA), anti-ERα (F-10) (WB 1:200, Cat. sc-8002, Santa Cruz Biotechnology), anti-Notch1 (C20) (WB 1:200, IP 6 μg, Cat. sc-6014-R, Santa Cruz Biotechnology), anti-Notch2 (WB 1:200, Cat. ab8926, Abcam, Cambridge, UK), anti-Notch1 ICD (Val1744) (WB 1:200, Cat. 2421, Cell Signaling, Danvers, MA, USA), anti-Notch2 ICD (WB 1:200, Cat. ab52302, Abcam), anti-Hey1 (WB 1:1000, Cat. ab154077, Abcam), anti-β-actin (AC-15) (WB 1:15000, Cat. ab6276, Abcam), anti-β-actin (WB 1:1000, Cat. 4967, Cell Signaling), anti-GAPDH (WB 1:20000, Clone GAPDH-71.1, Cat. G8795, Sigma-Aldrich), anti-HDAC2 (Ab-394) (WB 1:1000, Cat. SAB4300412, Sigma-Aldrich), anti-DsRed (WB 1:500, Cat. 632496, Takara Bio, San Jose, CA, USA), anti-GFP (GF28R) (WB 1:1000, Cat. MA5-15256, Thermo Fisher Scientific), anti-Cyclin A2 (BF683) (WB 1:1333, Cat. 4656, Cell Signaling), anti-Cyclin B1 (V152) (WB 1:1333, Cat. 4135, Cell Signaling), Goat anti-Mouse IgG (WB 1:4000, Cat. A4416, Sigma-Aldrich), and Goat anti-Rabbit IgG (WB 1:4000, Cat. A6667, Sigma-Aldrich). Image acquisition and densitometric analysis of the gels, blots, and film were performed with Bio-Rad Image Lab software V4.0.1 (Bio-Rad, Hercules, CA, USA).

### Immunoprecipitation, recombinant proteins, and pulldown assays

Immunoprecipitation, generation and purification of recombinant proteins, and pulldown assays were performed as described previously^39^. Briefly, mouse organs, cultured cells, and *Escherichia coli* BL21 (DE3) pLysS (Cat. 44-0034, Invitrogen) expressing GST-tagged proteins were homogenized or sonicated in lysis buffer A (10 mM HEPES, pH 7.4, 100 mM NaCl, 2 mM EDTA, 1 mM 2-mercaptoethanol, 0.5% Triton X-100) with a protease inhibitor cocktail tablet (Cat. 11836170001, Roche Diagnostics GmbH). The *E. coli* BL21 (DE3) pLysS expressing GIT1-His was sonicated in lysis buffer B (20 mM Tris-HCl, pH 8.0, 500 mM NaCl, 1 mM 2-mercaptoethanol, 0.1% Triton X-100) with a protease inhibitor cocktail tablet (Cat. 11836170001, Roche Diagnostics GmbH). Lysates were centrifuged at 20,000 g for 30 min at 4 °C. The supernatant with GIT1-His was incubated with a 1/10 volume of a 1:1 slurry of NI-NTA agarose (Invitrogen), washed using buffer B, eluted using 500 mM imidazole, and dialyzed in buffer A. The other supernatants were directly used for the next assay. For immunoprecipitation, 1 ml of supernatant was incubated with 6 μg of each antibody for 1 h at 4 °C, and then, 36 μl of a 1:1 slurry of Protein G Sepharose 4 Fast Flow (Cat. 17061801, Amersham Biosciences, Amersham, UK) was added and left to incubate overnight. The spin-down complex was washed using buffer C (buffer A + 50 mM NaCl) three times and solubilized in 60 μl of 2x SDS-PAGE buffer. For the pulldown assay, 1 ml of supernatants with GST–tagged proteins were incubated with 36 μl of a 1:1 slurry of glutathione-Sepharose 4B (Cat. 17075601, Amersham Biosciences) at 4 °C for 2 h and washed using buffer A three times. The spin-down complex was incubated with 1 ml of cell supernatants or purified GIT1-His overnight, washed using buffer C three times, and solubilized in 60 μl of 2x SDS-PAGE buffer.

### Plasmids, small interfering RNAs, small hairpin RNAs, and lentiviruses

GST-N1ICD constructs were created by subcloning PCR fragments of the mouse Notch 1 receptor into pGEX-6P1 (Amersham Biosciences). Construction of mRFP and GIT1-mRFP for transfection was carried out as described previously^39^. GIT1-His was made by subcloning PCR fragments of mouse GIT1 into pET-23a(+) (Cat. 69771, Sigma-Aldrich). Dominant-negative MAML1 was created by inserting a PCR fragment representing the N-terminal 13-74 amino acids of human MAML1 into the pcDNA4-mRFP plasmid^39^. Notch1ΔE−EGFP^56^ and the establishment of Notch1ΔE-GVP and MU100-Luc^28^ have been described previously. Site-directed mutants were generated using Pfu Turbo DNA Polymerase (Cat. 600250, Agilent Technologies, Santa Clara, CA, USA) with the following primers (underline indicates mutated sequences): mutNLS1 in GST-RAM/N-mutNLS1+2: 5′-GCCCCTGGGATCCGCCGCGGCCGCGGCGCAGCATGGCCAG-3′; mutNLS2 in GST-RAM/N-mutNLS1+2: 5′-GTGTCAGAGGCCAGCGCGGCGGCGGCGGCAGAGCCCCGTCGAC-3′; mutNLS1 in Notch1ΔE-mutNLS1+2−EGFP: 5′-GGTGCTGCTGTCCGCCGCGGCCGCGGCGGCCAAGCTACTG-3′; mutNLS2 in both Notch1ΔE-mutNLS1+2−EGFP and Notch1ΔE-mutNLS1+2−GVP: 5′-GTGTCAGAGGCCAGCGCGGCGGCGGCGGCAGAGCCCCTCGGCG-3′; mutNLS1 in Notch1ΔE-mutNLS1+2-GVP: 5′-GGTGCTGCTGTCCGCCGCGGCCGCGGCGGCCAAGCTACTG-3′; mutNLS3 in Notch1ΔE-mutNLS3+4−EGFP and Notch1ΔE-mutNLS1+2+3+4−EGFP: 5′-GGAGACGAAGACCTGGAGACCGCCGCATTCGCCTTTGAGGAGCCAGTAGTTCTCC-3′; mutNLS4 in Notch1ΔE-mutNLS3+4−EGFP and Notch1ΔE-mutNLS1+2+3+4−EGFP: both 5′-CTCAAGTCTGCCACACAGGGCGCCGCTGCCGCCGCACCCAGCACCAAAGGGCTGGC-3′ and 5′-GCAAGGAAGCTAAGGACCTCGCCGCAGCCGCTGCCGCCTCCCAGGATGGCAAGGGCTGCC-3′. Transfection of pRL-TK vector (Cat. E2241, Promega, Madison, WI, USA), GIT1 siRNA (Cat. HSS178932, Invitrogen), control siRNA (Cat. 45–2001, Invitrogen), GIT1 shRNA (Cat. sc-35477-SH, Santa Cruz Biotechnology), and control shRNA (Cat. sc-108066, Santa Cruz Biotechnology) was performed as described previously^39^. Lentiviral transduction was performed with control shRNA (LV-Ctrl-shRNA, Cat. SHC002V, Sigma-Aldrich) and GIT1 shRNAs (LV-GIT1-shRNA3, Cat. TRCN0000008401; LV-GIT1-shRNA4, Cat. TRCN0000008403; Sigma-Aldrich) for one day. mRFP and mRFP-GIT1 lentiviral transduction particles were produced using the lentiviral package system: pCDF1-MC2_EF1Puro (Cat. CD110B-SBI, BioCat GmbH, Heidelberg, Germany), p.MD2.G (Cat. 12259, AddGene, Watertown, MA, USA), and ps.PAX2 (Cat. 12260, AddGene).

### Oestrogen regulated GIT1 expression assay

For oestrogen experiments, cells were starved for two days in phenol red-free RPMI-1640 (Cat. 11835030, Invitrogen) plus 20 μg/ml insulin (Cat. 12585014, Invitrogen) for BT474, HCC1954, and MCF7 cells or in phenol red-free or DMEM (Cat. 31053028, Invitrogen) plus 1% L-glutamine (Cat. 25030081, Invitrogen) for MDA-MB-231 and MDA-MB-361 cells, together with 10% charcoal-stripped FBS (Cat. 12676011, Invitrogen) and 0.5–1.0% penicillin-streptomycin (Cat. 15140122, Invitrogen). The cells were then treated with 10 nM 17β-oestradiol (E2, Cat. E2758, Sigma-Aldrich) and/or with 1 μM fulvestrant (Cat. I4409, Sigma-Aldrich) or 1 μM 4-hydroxytamoxifen (Cat. H6278-10MG, Sigma-Aldrich or Cat. ALX-550-361-M001, Enzo Life Sciences, Farmingdale, NY, USA) for three days. The medium was changed every day. The cells were collected using lysis buffer and subjected to SDS-PAGE and western blotting for the indicated proteins.

### Fluorescence immunohistochemistry and confocal microscopy

Immunohistochemistry was carried out, with modifications, as previously described^21^. Briefly, tissue sections were cut from paraffin blocks and dewaxed twice in xylene for 5 min, dehydrated in an alcohol gradient (100%, 95%, and 85%), and subjected to antigen retrieval in 10 mM sodium citrate buffer (pH 6.0, Cat. Dako S1699, Agilent Technologies) by heating it twice in a microwave oven using half power for 8 min. Nonspecific binding was avoided by incubating with blocking buffer (5% skim milk, 0.1% Triton X-100, 0.1% Tween in PBS) for 1 h at RT in a humidified chamber. Sections were incubated overnight with primary antibodies diluted in blocking buffer. After the sections were washed in PBS, they were incubated with corresponding secondary antibodies for 1 h at RT and then washed once more in PBS. The following antibodies were used: anti-GIT1 (1:1000, Cat. N39B/8, NeuroMab), anti-ERα (HC20) (1:150, Cat. sc-543, Santa Cruz Biotechnology), anti-Notch1 ICD (1:100, Cat. ab8925, Abcam), anti-ALDH1A1 (EP1933Y) (1:20, Cat. ab52492, Abcam), Goat anti-Mouse IgG Alexa555 (1:1000, Cat. A-21425, Thermo Fisher Scientific), and Goat anti-Rabbit IgG Alexa488 (1:1000, Cat. A-11034, Thermo Fisher Scientific). Immunostaining of xenograft tumours with stable cells expressing GIT1-mRFP showed no mRFP signal since the fluorescence had faded during the selection process of stable colonies. Immunostained sections or EGFP-expressing cells were mounted using VectaShield with DAPI (Cat. H-1500, Vector Laboratories, Burlingame, CA, USA) and scanned with confocal microscopes Olympus FluoView1000 (Olympus, Tokyo, Japan) or Zeiss LSM780 (Carl Zeiss, Jena, Germany) and Olympus FV10-ASW software or Zeiss Zen Black V2.1 software, respectively. Fluorescence was quantified with ImageJ V2.0.0-rc-43/1.52n software (NIH, Washington, DC, USA). GIT1 intensities were normalized to DAPI.

### Reporter gene analysis

For analysis of GIT1-regulated Notch signalling, 1.35 × 10^5^ MDA-MB-231 cells/well were seeded in 6-well plates for one day and then transfected with 800 ng of RBP-J*κ* Reporter kit (Cat. CCS-014L, Qiagen, Hilden, Germany, a mixture of RBP-J*κ*-responsive firefly luciferase reporter and an internal control construct constitutively expressing Renilla luciferase) combined with 2.5 μM of GIT1 siRNA/control siRNA or 600 ng GIT1-mRFP/mRFP using 4 μl of Lipofectamine 2000 (Cat. 11668-019, Invitrogen). Each well with transfected cells was reseeded in three wells coated with immobilized Jagged1 in a 24-well-plate. For GIT1-regulated Notch ICD nuclear distribution, 2.0 ×10^4^ MDA-MB-231 cells/well were seeded in a 24-well plate one day before and transfected with 100 ng of Notch1ΔE-GVP^28^ or Notch1ΔE-mutNLS1+2−GVP together with 200 ng of MH1000-Luc plus 20 ng of pRL-TK (Cat. E2241, Promega, Madison, WI, USA) with or without 0.5 μM GIT1 siRNA/control siRNA or 120 ng GIT1-mRFP/mRFP using 1 μl of Lipofectamine 2000 (Cat. 11668019, Thermo Fisher Scientific). DAPT for control wells was added 6 h after transfection. Luciferase activities were assayed two days after transfection using the Dual-Luciferase Reporter Assay System (Cat. E1910, Promega) following the manufacturer’s protocol.

### Real-time quantitative reverse transcription-PCR analysis

RNA was isolated using TRIzol^®^ Reagent, and cDNA was produced using SuperScript II Reverse Transcriptase (Cat. 18064-014, Invitrogen) and random primers (Cat. 48190-011, Invitrogen). Primers for the qPCR analysis of Hey1 (forward: 5′-CGAGCTGGACGAGACCAT-3′, and reverse: 5′-GAGCCGAACTCAAGTTTCCA-3′) were designed using Primer Express. qPCR experiments were performed using SYBR Green Master Mix (Cat. 4309155, Applied Biosystems, Foster City, CA, USA) with 10 μM of forward/reverse primers and analysed in real-time using the 7900 HT Fast Real Time PCR system with SDS 2.3 software (Applied Biosystems). The relative quantity of Hey1 expression levels were calculated based on the qPCR analysis with Quantum RNA Universal 18 S primers (Ambion, Invitrogen) and were normalized to one.

### Flow cytometry

Flow cytometric analysis of ALDH and Hey1 activity in single cells was performed using a FACSVantage flow cytometer (BD Biosciences, San Diego, CA, USA). For analysis of the ALDH enzymatic activity, an ALDEFLUOR kit (Cat. 1700, StemCell Technologies, Cambridge, UK) was used following the manufacturer’s protocol. Briefly, stably transfected cells were suspended in ALDEFLUOR assay buffer containing the ALDH substrate (BAAA, 1.5 μM/1 ×10^6^ cells) and incubated for 50 min at 37 °C. Each BAAA-treated cell sample was equally aliquoted for a negative control by treatment with 15 mM diethylaminobenzaldehyde (DEAB), a specific ALDH inhibitor. For single-cell analysis of xenografts, tumours were dissociated using the FFPE Tissue Dissociation Kit (Cat. 130-118-052, Miltenyi Biotec, Bergisch Gladbach, Germany) following the manufacturer’s protocol. Dissociated cells were then incubated with primary antibodies or control IgG at RT for 1 h, followed by the Alexa Fluor 488-conjugated secondary antibody at RT for 1 h. The sorting gates were established using the negative controls. Data were analysed using CellQuest Pro V6.0 software (BD Biosciences) and were normalized to one.

### Clonogenic, spheroid, and proliferation analyses

For the clonogenic assay, with 150 cells/well, MDA-MB-231 or HCC1395 breast cancer cells were seeded in a 6-well plate and cultured in DMEM or RPMI 1640 with 10% FBS without or with DAPT and specific antibiotics to maintain stably transfected cells. The medium was changed every 2-3 days until colonies of at least 50 cells were formed (approximately 10 days). The cells were then fixed with PFA and stained with crystal violet (Cat. C0775, Sigma-Aldrich). The colonies with more than 50 cells were counted manually in a stereomicroscope. The percentage of colonies per well was calculated as the number of colonies divided by the number of seeded cells × 100%. For the spheroid assay, cells were collected, counted, and diluted to 1 cell, 2 cells, 3 cells, or 5 cells per drop (25 μl of medium). Approximately 30 drops per experiment were then seeded on the inverted lid of a cell culture dish (Cat. 08-772E, Thermo Fisher Scientific). The lid with hanging drops of cells was thereafter placed on the PBS-filled (10 ml) bottom chamber. Spheroid formation was assessed using a bright field microscope. The proliferation assay was analysed using 5-ethynyl-2′-deoxyuridine (EdU, Cat. E10187, Invitrogen) staining. For in vitro applications, cells were treated with 10 μM EdU at 37 °C in an incubator for 1 h. For in vivo applications, xenografted mice were intraperitoneally injected with 0.64 mg EdU in 100 μL of PBS per 10 grams of mouse body weight one day before the mice were killed. Detection of EdU was performed according to the manufacturer’s protocol.

### Animal studies

Animal experiments were performed in accordance with protocols approved by The Northern Stockholm Animal Ethical Committee (Ethical numbers: N391/11, N231/14). The mice were housed in standard cages in a temperature- and humidity-controlled environment with 12 h light/12 h dark cycles and *ad libitum* access to food and water (R36, R70 from Lantmännen, Sweden) and were cared for in accordance with Swedish national regulations (SFS 1988: 534, SFS 1988: 539 and SFS 1988: 541). Six-week-old female NMRI nu/nu mice (Scanbur, Stockholm, Sweden) were randomly assigned to groups of ten animals, and stably transfected MDA-MB-231 or HCC1395 cells suspended in DMEM (MDA-MB-231) or RPMI-1640 (HCC1395) medium with neither FBS nor antibiotics were bilaterally (Fig. 5a) or unilaterally (Fig. 5b–f, k) subcutaneously injected in the right/left rear flank. The number of cells per injection was 1 × 10^7^ (MDA-MB-231) or 5 × 10^6^ (HCC1395). The animals were weighed, and tumours were measured with digital calipers at least twice per week. A tumour was defined as >100 mm^3^ (MDA-MB-231) and >500 mm^3^ (HCC1395). The tumour volume was calculated using the formula length × width^2^ × 0.44^57,58^. The initial tumour growth rate was analysed using a regression model based on exponential (Malthusian) growth *G*(*t*) Eq. (1) at time *t* (days):

1 $$G\left(t\right)={G}_{0}{{{{{{\rm{e}}}}}}}^{{kt}}$$

where *G*_0_ denotes the tumour volume at *t* = 0 and *k* is the growth rate. Time was days from the tumour volume >100 mm^3^, and the doubling time was calculated as ln(2)/*k*. No animals were excluded from the statistical analysis, but when we compared days until tumour appearance, only mice that had tumours could be included. No differences in food intake, body weight, or signs of toxicity were observed between the different animal groups. When the tumours reached 500 mm^3^ (MDA-MB-231 cells) or 1000 mm^3^ (HCC1395 cells) or at signs at discomfort according to the protocol, the animal was killed, and the tumour was removed, weighed, and fixed for further experiments. In the case of bilateral injections, the animal was killed when one of the two injected tumours reached 500 mm^3^. Gross examinations of the animals’ body condition were performed continuously and at signs of bite marks or wounds, the experiment was terminated.

### Patient data analysis

Survival analysis was performed in the TCGA Firehose Legacy dataset^59^ (*n* = 1092) using the survival R package (http://CRAN.R-project.org/package=survival/) for Cox regression analysis and the survplot R package (http://www.cbs.dtu.dk/~eklund/survplot/) for generating the Kaplan–Meier plot. Primary tumour samples from breast cancer patients who had been followed up for <10 years were stratified into high (top 20%) and low (bottom 20%) GIT1 expression.

Analysis of GIT1 protein levels (mass spectrometry standardized Z-scores) was performed using data (Clinical Proteomic Tumour Analysis Consortium, CPTAC) obtained from the TCGA Firehose Legacy dataset. Patient samples with 1–10% ER(+) status were excluded from the ER(−) group since international guidelines state that a threshold of 1% should be used for allocating patients into negative and positive expression subgroups^55^.

Differential gene expression analysis was performed in the METABRIC dataset (*n* = 2509) using the limma R V3.32.7^60^ package (https://bioconductor.org/packages/limma/). The illuminaHumanv3.db annotation package was used to assess the microarray gene probes between high (top 20%) and low (bottom 20%) GIT1-expressing breast cancer patients.

Kaplan–Meier analysis of correlations between GIT1 expression and the relapse-free survival of breast cancer patients (*n* = 4929) with ER(+) and/or ER(−) was performed using Kaplan–Meier plotter (KMplot, http://www.kmplot.com)^61^. Breast cancer patients were divided into high and low GIT1 expression groups based on trichotomization (lower tercile (T1) versus upper tercile (T3)). The probe 218030_at was used for all analyses, and the ER status was array based^62^.

### Statistics

All quantitative data were collected from experiments performed in at least triplicate and expressed as the mean ± s.e.m. The statistical test used and the definition *n* for each analysis are listed in the figure legends. In the text, *P*-values and replicates *n* refer to unpaired *t* tests and independent biological replicates, respectively, unless stated otherwise. For comparisons between two groups, two-sided unpaired *t* tests (parametric) or Mann–Whitney *U*-tests (nonparametric) were used. For comparisons across more than two groups, ordinary one-way ANOVA was used, and where significance was detected, a Tukey’s post hoc comparison was performed. For comparisons between Kaplan–Meier curves, log-rank tests were used, with correction for multiple comparisons. For comparisons between regression coefficients, extra-sum-of-squares *F*-tests were used. Tests involving correlations were performed using Spearman’s method. No statistical methods were used to predetermine the sample size. Statistical tests were performed in R V4.0.2 software (A language and environment for statistical computing. R Foundation for Statistical Computing, Vienna, Austria, URL http://www.R-project.org/), Microsoft Excel V16 software (Microsoft Corporation, Redmond, WA, USA), and GraphPad Prism V8/9 software (GraphPad Software, San Diego, CA, USA). Differences were considered significant at *P < 0.05, **P < 0.01, ***P < 0.005, and ****P < 0.001, and not significant (ns) at *P* ≥ 0.05. The experiments were not randomized, and investigators were not blinded to allocation during experiments and outcome assessment.

### Reporting summary

Further information on research design is available in the Nature Research Reporting Summary linked to this article.

## Supplementary information

Supplementary Information

Reporting Summary

## Data Availability

A reporting summary for this article is available as Supplementary Information file. The publicly available breast cancer datasets (TCGA (Firehose Legacy) and METABRIC) used in this study are available via cBioPortal [http://www.cbioportal.org]. Accession codes for publicly available breast cancer data sets that were analysed with KM-plotter^61^ are listed in Supplementary Table 1. The main data supporting the findings of this study are available within the article and its Supplementary Figures. The source data underlying Figs. 1–5, Supplementary Figs 1–3, and Supplementary Figs 5–13 are provided as a Source Data file. All the other data are available within the article and its Supplementary Information. Source data are provided with this paper.

## Source data

Source Data

## References

[1] Ferlay J (2010). Estimates of worldwide burden of cancer in 2008: GLOBOCAN 2008. Int. J. Cancer.

[2] Andersson ER, Lendahl U (2014). Therapeutic modulation of Notch signalling—are we there yet?. Nat. Rev. Drug Discov..

[3] Lafkas D (2015). Therapeutic antibodies reveal Notch control of transdifferentiation in the adult lung. Nature.

[4] Wu Y (2010). Therapeutic antibody targeting of individual Notch receptors. Nature.

[5] Dickson BC (2007). High-level JAG1 mRNA and protein predict poor outcome in breast cancer. Mod. Pathol..

[6] Pece S (2004). Loss of negative regulation by Numb over Notch is relevant to human breast carcinogenesis. J. Cell Biol..

[7] Reedijk M (2005). High-level coexpression of JAG1 and NOTCH1 is observed in human breast cancer and is associated with poor overall survival. Cancer Res..

[8] Stylianou S, Clarke RB, Brennan K (2006). Aberrant activation of notch signaling in human breast cancer. Cancer Res..

[9] Rizzo P (2008). Cross-talk between notch and the estrogen receptor in breast cancer suggests novel therapeutic approaches. Cancer Res..

[10] Simoes BM (2015). Anti-estrogen resistance in human breast tumors is driven by JAG1-NOTCH4-dependent cancer stem cell activity. Cell Rep..

[11] Lee CW, Raskett CM, Prudovsky I, Altieri DC (2008). Molecular dependence of estrogen receptor-negative breast cancer on a notch-survivin signaling axis. Cancer Res..

[12] Speiser J (2012). Notch-1 and Notch-4 biomarker expression in triple-negative breast cancer. Int J. Surg. Pathol..

[13] Yao K (2011). Notch-1 and notch-4 receptors as prognostic markers in breast cancer. Int J. Surg. Pathol..

[14] Kopan R, Ilagan MX (2009). The canonical Notch signaling pathway: unfolding the activation mechanism. Cell.

[15] Siebel C, Lendahl U (2017). Notch signaling in development, tissue homeostasis, and disease. Physiol. Rev..

[16] Huenniger K (2010). Notch1 signaling is mediated by importins alpha 3, 4, and 7. Cell Mol. Life Sci..

[17] Jeffries S, Capobianco AJ (2000). Neoplastic transformation by Notch requires nuclear localization. Mol. Cell Biol..

[18] Dent LG (2015). The GTPase regulatory proteins Pix and Git control tissue growth via the Hippo pathway. Curr. Biol..

[19] Liu J, Zeng L, Kennedy RM, Gruenig NM, Childs SJ (2012). betaPix plays a dual role in cerebral vascular stability and angiogenesis, and interacts with integrin alphavbeta8. Dev. Biol..

[20] Zhou W, Li X, Premont RT (2016). Expanding functions of GIT Arf GTPase-activating proteins, PIX Rho guanine nucleotide exchange factors and GIT-PIX complexes. J. Cell Sci..

[21] Jin S (2013). Non-canonical Notch signaling activates IL-6/JAK/STAT signaling in breast tumor cells and is controlled by p53 and IKKalpha/IKKbeta. Oncogene.

[22] Andrieu G, Tran AH, Strissel KJ, Denis GV (2016). BRD4 regulates breast cancer dissemination through Jagged1/Notch1 signaling. Cancer Res..

[23] Shen Q (2017). Notch Shapes the Innate Immunophenotype in Breast Cancer. Cancer Discov..

[24] Sahlgren C, Gustafsson MV, Jin S, Poellinger L, Lendahl U (2008). Notch signaling mediates hypoxia-induced tumor cell migration and invasion. Proc. Natl Acad. Sci. USA.

[25] Badve S, Nakshatri H (2012). Breast-cancer stem cells-beyond semantics. Lancet Oncol..

[26] Li W (2017). Unraveling the roles of CD44/CD24 and ALDH1 as cancer stem cell markers in tumorigenesis and metastasis. Sci. Rep..

[27] Miranda A (2019). Cancer stemness, intratumoral heterogeneity, and immune response across cancers. Proc. Natl Acad. Sci. USA.

[28] Karlstrom H, Bergman A, Lendahl U, Naslund J, Lundkvist J (2002). A sensitive and quantitative assay for measuring cleavage of presenilin substrates. J. Biol. Chem..

[29] Nam Y, Sliz P, Song L, Aster JC, Blacklow SC (2006). Structural basis for cooperativity in recruitment of MAML coactivators to Notch transcription complexes. Cell.

[30] Weng AP (2003). Growth suppression of pre-T acute lymphoblastic leukemia cells by inhibition of notch signaling. Mol. Cell Biol..

[31] Braune EB, Lendahl U (2016). Notch—a goldilocks signaling pathway in disease and cancer therapy. Discov. Med..

[32] Koch U, Radtke F (2010). Notch signaling in solid tumors. Curr. Top. Dev. Biol..

[33] Robinson DR (2011). Functionally recurrent rearrangements of the MAST kinase and Notch gene families in breast cancer. Nat. Med..

[34] Chang JS (2015). GIT1 promotes lung cancer cell metastasis through modulating Rac1/Cdc42 activity and is associated with poor prognosis. Oncotarget.

[35] Huang WC (2014). miRNA-491-5p and GIT1 serve as modulators and biomarkers for oral squamous cell carcinoma invasion and metastasis. Cancer Res..

[36] Peng H (2013). MAT2B-GIT1 interplay activates MEK1/ERK 1 and 2 to induce growth in human liver and colon cancer. Hepatology.

[37] Weaver AN (2016). Notch signaling activation is associated with patient mortality and increased FGF1-mediated invasion in squamous cell carcinoma of the oral cavity. Mol. Cancer Res..

[38] Chan SH (2014). MicroRNA-149 targets GIT1 to suppress integrin signaling and breast cancer metastasis. Oncogene.

[39] Zhang S, Hisatsune C, Matsu-Ura T, Mikoshiba K (2009). G-protein-coupled receptor kinase-interacting proteins inhibit apoptosis by inositol 1,4,5-triphosphate receptor-mediated Ca^2+^ signal regulation. J. Biol. Chem..

[40] Alim I (2019). Selenium drives a transcriptional adaptive program to block ferroptosis and treat stroke. Cell.

[41] Smithson LJ, Gutmann DH (2016). Proteomic analysis reveals GIT1 as a novel mTOR complex component critical for mediating astrocyte survival. Genes Dev..

[42] Li L (2019). GIT1 regulates angiogenic factor secretion in bone marrow mesenchymal stem cells via NF-kappaB/Notch signalling to promote angiogenesis. Cell Prolif..

[43] Majumder S (2016). G-protein-coupled receptor-2-interacting protein-1 controls stalk cell fate by inhibiting delta-like 4-notch1 signaling. Cell Rep..

[44] Andersson ER, Sandberg R, Lendahl U (2011). Notch signaling: simplicity in design, versatility in function. Development.

[45] Bray SJ (2006). Notch signalling: a simple pathway becomes complex. Nat. Rev. Mol. Cell Biol..

[46] Landor SK (2011). Hypo- and hyperactivated Notch signaling induce a glycolytic switch through distinct mechanisms. Proc. Natl Acad. Sci. USA.

[47] Mazzone M (2010). Dose-dependent induction of distinct phenotypic responses to Notch pathway activation in mammary epithelial cells. Proc. Natl Acad. Sci. USA.

[48] Gupta-Rossi N (2004). Monoubiquitination and endocytosis direct gamma-secretase cleavage of activated Notch receptor. J. Cell Biol..

[49] Sjoqvist M (2014). PKCzeta regulates Notch receptor routing and activity in a Notch signaling-dependent manner. Cell Res..

[50] Santio, N. M. et al. Phosphorylation of Notch1 by Pim kinases promotes oncogenic signaling in breast and prostate cancer cells. *Oncotarget*10.18632/oncotarget.9215 (2016). 10.18632/oncotarget.9215 PMC5190019 27281612

[51] Ginestier C (2007). ALDH1 is a marker of normal and malignant human mammary stem cells and a predictor of poor clinical outcome. Cell Stem cell.

[52] Liu Y (2014). ALDH1A1 expression correlates with clinicopathologic features and poor prognosis of breast cancer patients: a systematic review and meta-analysis. BMC Cancer.

[53] Zhao D (2014). NOTCH-induced aldehyde dehydrogenase 1A1 deacetylation promotes breast cancer stem cells. J. Clin. Invest..

[54] Bhaskaran N (2009). Comparative proteome profiling of MCF10A and 184A1 human breast epithelial cells emphasized involvement of CDK4 and cyclin D3 in cell proliferation. Proteom. Clin. Appl..

[55] Coates AS (2015). Tailoring therapies–improving the management of early breast cancer: St Gallen International Expert Consensus on the Primary Therapy of Early Breast Cancer 2015. Ann. Oncol..

[56] Das D (2010). Notch induces cyclin-D1-dependent proliferation during a specific temporal window of neural differentiation in ES cells. Dev. Biol..

[57] Tomayko MM, Reynolds CP (1989). Determination of subcutaneous tumor size in athymic (nude) mice. Cancer Chemother. Pharmacol..

[58] Wassberg E, Hedborg F, Skoldenberg E, Stridsberg M, Christofferson R (1999). Inhibition of angiogenesis induces chromaffin differentiation and apoptosis in neuroblastoma. Am. J. Pathol..

[59] Cancer Genome Atlas, N. (2012). Comprehensive molecular portraits of human breast tumours. Nature.

[60] Ritchie ME (2015). limma powers differential expression analyses for RNA-sequencing and microarray studies. Nucleic Acids Res..

[61] Gyorffy B (2010). An online survival analysis tool to rapidly assess the effect of 22,277 genes on breast cancer prognosis using microarray data of 1,809 patients. Breast Cancer Res. Treat..

[62] Gyorffy B (2012). RecurrenceOnline: an online analysis tool to determine breast cancer recurrence and hormone receptor status using microarray data. Breast Cancer Res. Treat..

[63] Curtis C (2012). The genomic and transcriptomic architecture of 2,000 breast tumours reveals novel subgroups. Nature.

