# Supplementary material for: GIT1 protects against breast cancer growth through negative regulation of Notch
Source: Nat Commun. 2022 Mar 22;13:1537. doi: 10.1038/s41467-022-28631-y (PMC8940956; doi:10.1038/s41467-022-28631-y)
Supplement: Supplementary file 1 — Supplementary Information [file 41467_2022_28631_MOESM1_ESM.pdf]

# GIT1 protects against breast cancer growth through negative regulation of Notch

Songbai Zhang[1], Ayako Miyakawa[1,2], Malin Wickström[3], Cecilia Dyberg[3], Lauri Louhivuori[1], Manuel Varas-Godoy[1,4], Kati Kemppainen[5,6], Shigeaki Kanatani[1], Dagmara Kaczynska[1], Ivar Dehnisch Ellström[1], Lotta Elfman[3], Pauliina Kronqvist[7], Heli Repo[7], Katsuhiko Mikoshiba[8,9,10], Cecilia Sahlgren[5,6,11], John Inge Johnsen[3], and Per Uhlén[1]

**Affiliations:**

[1] Department of Medical Biochemistry and Biophysics, Karolinska Institutet, Stockholm, Sweden.
[2] Department of Molecular Medicine and Surgery, Karolinska University Hospital, Stockholm, Sweden.
[3] Department of Women's and Children's Health, Karolinska Institutet, Stockholm, Sweden.
[4] Centro de Biología Celular y Biomedicina (CEBICEM), Facultad de Medicina y Ciencia, Universidad San Sebastián, Santiago, Chile.
[5] Turku Bioscience, Åbo Akademi University and University of Turku, Turku, Finland.
[6] Faculty of Science and Engineering, Åbo Akademi University, Turku, Finland.
[7] Department of Pathology, University of Turku, Turku, Finland.
[8] Shanghai Institute for Advanced Immunochemical Studies, ShanghaiTech University, Shanghai, China.
[9] RIKEN Center for Life Science Technologies (CLST), Chuo-ku, Kobe, Japan.
[10] Department of Biomolecular Science, Faculty of Science, Toho University, Chiba, Japan.
[11] Institute for Complex Molecular Systems, Eindhoven University of Technology, Eindhoven, The Netherlands.

# Supplementary Information

Supplementary Figure 1 .......... 2
Supplementary Figure 2 .......... 3
Supplementary Figure 3 .......... 4
Supplementary Figure 4 .......... 5
Supplementary Figure 5 .......... 6
Supplementary Figure 6 .......... 7
Supplementary Figure 7 .......... 8
Supplementary Figure 8 .......... 9
Supplementary Figure 9 .......... 10
Supplementary Figure 10 .......... 11
Supplementary Figure 11 .......... 12
Supplementary Figure 12 .......... 13
Supplementary Figure 13 .......... 14
Supplementary Table 1 .......... 15

# Supplementary Figures

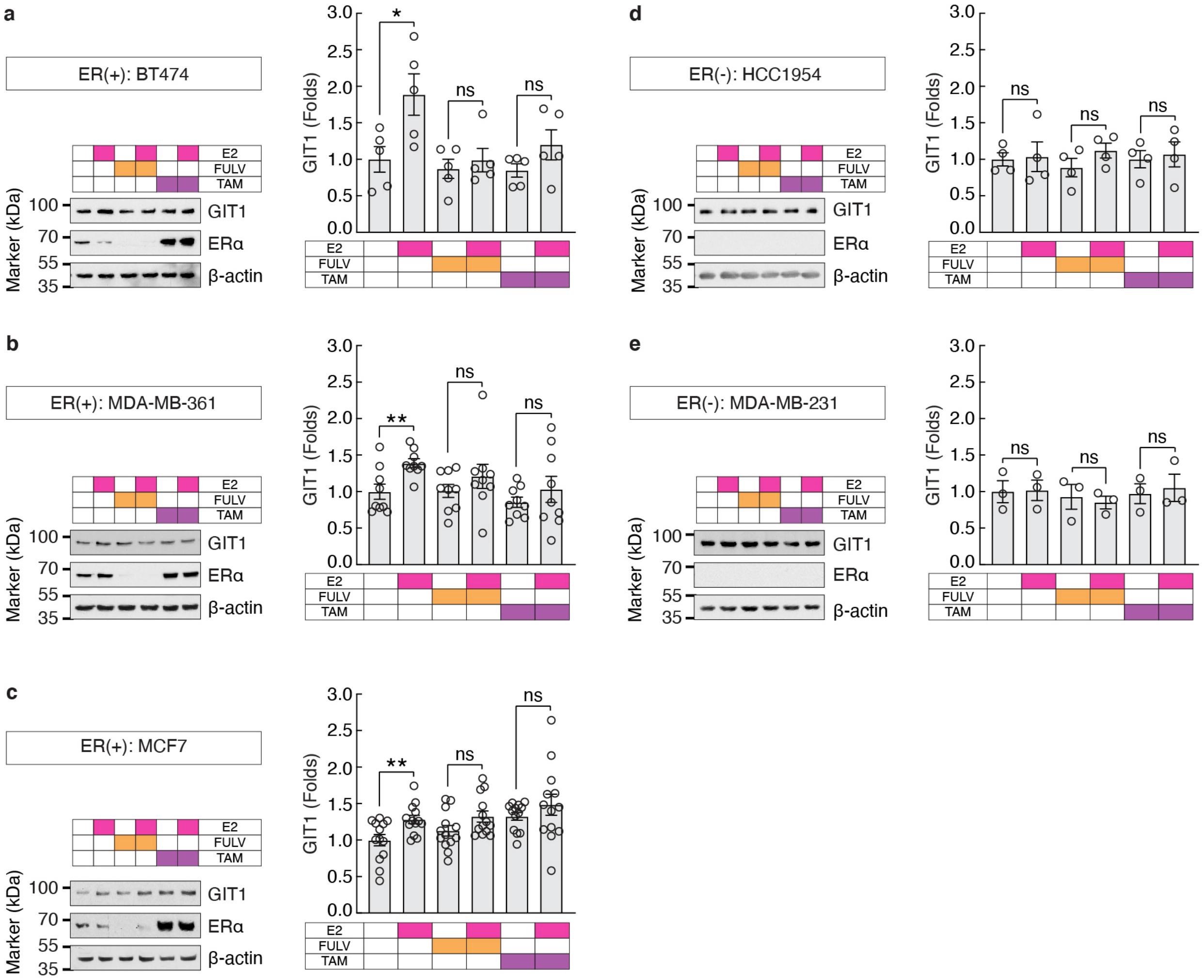


**Supplementary Figure 1 | Oestrogen alters the expression of GIT1 in ER(+) breast cancer cells. a-e**, Western blots and the quantitative analysis of GIT1 levels normalized to β-actin in ER(+) breast cancer cells BT474 (**a**; *n* = 5, Ctrl versus 17β-oestradiol (E2), *P* = 0.028; fulvestrant (FULV) versus FULV+E2, *P* = 0.58; tamoxifen (TAM) versus TAM+E2, *P* = 0.16; *t* tests), MDA-MB-361 (**b**; *n* = 9, Ctrl versus E2, *P* = 0.0056; FULV versus FULV+E2, *P* = 0.31; TAM versus TAM+E2, *P* = 0.37; *t* tests), and MCF7 (**c**; *n* = 13, Ctrl versus E2, *P* = 0.0077; FULV versus FULV+E2, *P* = 0.078; TAM versus TAM+E2, *P* = 0.31; *t* tests), and in ER(-) breast cancer cells HCC1954 (**d**; *n* = 4, Ctrl versus E2, *P* = 0.88; FULV versus FULV+E2, *P* = 0.20; TAM versus TAM+E2, *P* = 0.76; *t* tests) and MDA-MB-231 (**e**; *n* = 3, Ctrl versus E2, *P* = 0.93; FULV versus FULV+E2, *P* = 0.70; TAM versus TAM+E2, *P* = 0.74; *t* tests) treated with 1 μM fulvestrant (FULV) or 1 μM tamoxifen (TAM), two oestrogen receptor antagonists, or 10 nM 17β-oestradiol (E2) for three days. All data are shown as the mean ± s.e.m. **P* < 0.05, ***P* < 0.01, ns, not significant by two-sided unpaired *t* tests. Source data are provided as a Source Data file.

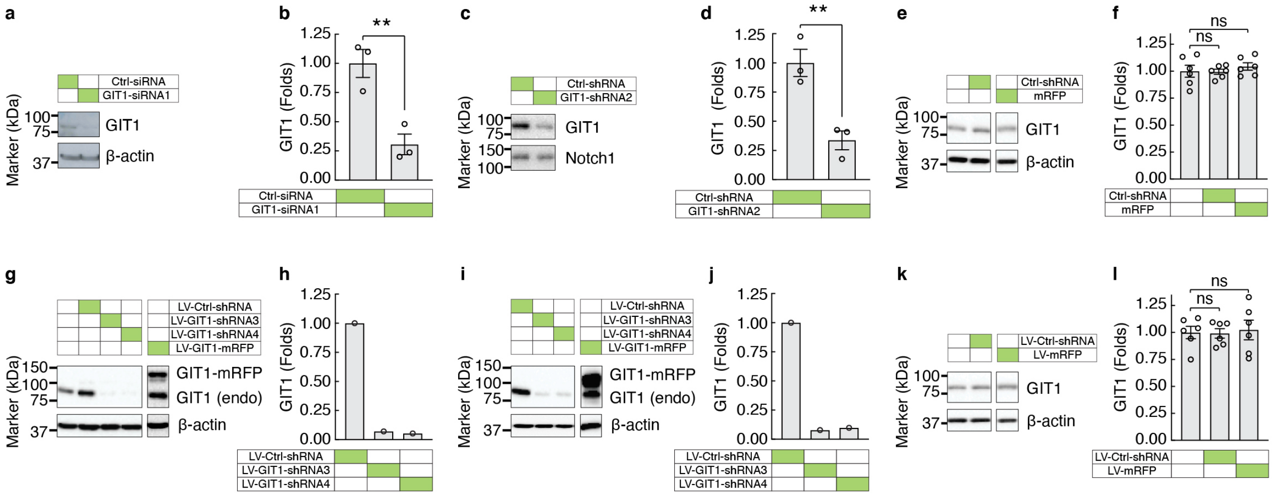


**Supplementary Figure 2 | Knockdown of GIT1 in breast cancer cells. a**-**f**, Western blots of GIT1 in MDA-MB-231 cells treated with GIT1-siRNA1 (**a**), GIT1-shRNA2 (**c**), or mRFP (**e**) and the quantitative analysis (**b**; GIT1-siRNA1, *n* = 3, *P* = 0.0095; **d**; GIT1-shRNA2, *n* = 3, *P* = 0.0095; **f**; *n* = 6, Ctrl-shRNA versus control, *P* = 0.93; mRFP versus control, *P* = 0.51; *t* tests). **g**-**l**, Western blots of GIT1 overexpression or knockdown in MDA-MB-231 cells (**g**,**h**) and HCC1395 cells (**i**-**l**) transduced with lentiviruses (LV) LV-GIT1-shRNA3, LV-GIT1-shRNA4, or LV-GIT1-mRFP (**g**, **i**) and LV-mRFP (**k**) and the quantitative analysis (**l**; *n* = 6, LV-Ctrl-shRNA versus control, *P* = 0.92; LV-mRFP versus control, *P* = 0.82; *t* tests). All data are shown as the mean ± s.e.m. *n* denotes the number of biologically independent replicates. ***P* < 0.01, ns, not significant by two-sided unpaired *t* tests. Source data are provided as a Source Data file.

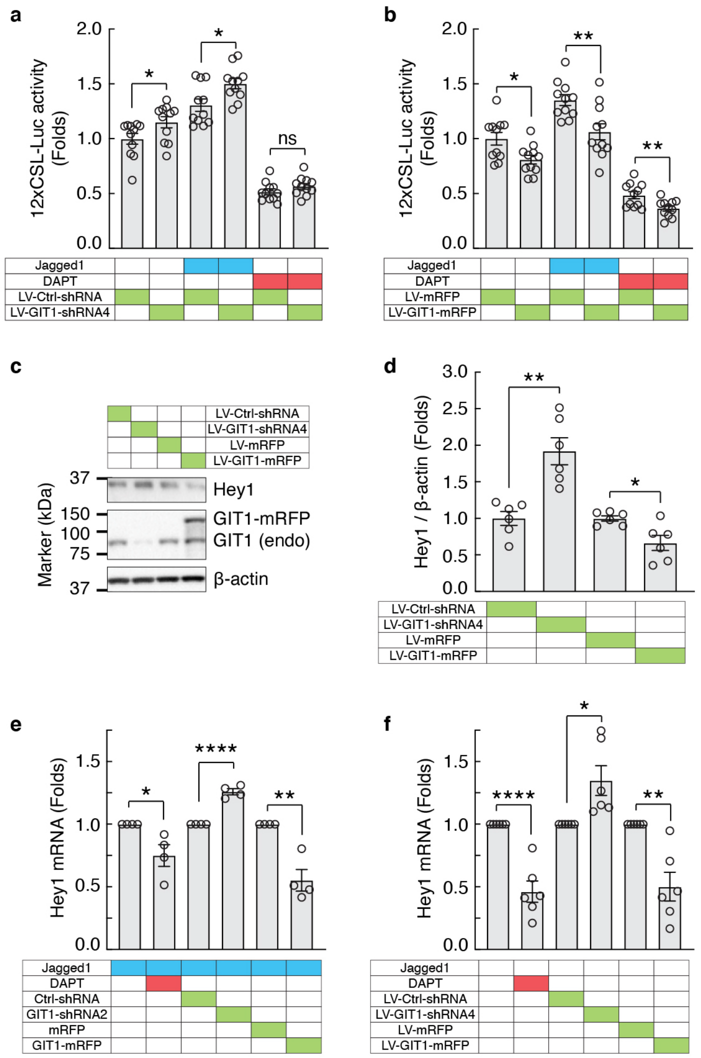


**Supplementary Figure 3 | GIT1 negatively regulates Notch signalling in breast cancer cells. a, b,** Luciferase reporter assay of 12xCSL-Luc in HCC1395 cells transduced with LV-GIT1-shRNA4 (**a**; *n* = 11, LV-Ctrl-shRNA versus LV-GIT1-shRNA4, *P* = 0.044; LV-Ctrl-shRNA+Jagged1 versus LV-GIT1-shRNA4+Jagged1, *P* = 0.014; LV-Ctrl-shRNA+DAPT versus LV-GIT1-shRNA4+DAPT, *P* = 0.18; *t* tests) or LV-GIT1-mRFP (**b**; *n* = 11, LV-mRFP versus LV-GIT1-mRFP, *P* = 0.013; LV-mRFP+Jagged1 versus LV-GIT1-mRFP+Jagged1, *P* = 0.0039; LV-mRFP+DAPT versus LV-GIT1-mRFP+DAPT, *P* = 0.0076; *t* tests) treated as indicated. **c**, **d**, Western blot of the Hey1 expression in HCC1395 cells treated as indicated (**c**) and the quantitative analysis (**d**; *n* = 6, LV-Ctrl-shRNA versus LV-GIT1-shRNA4, *P* = 0.0013; LV-mRFP versus LV-GIT1-mRFP, *P* = 0.013; *t* tests). **e**, **f**, Hey1 mRNA expression in MDA-MB-231 cells treated with (**e**; *n* = 4, Ctrl versus DAPT, *P* = 0.028; Ctrl-shRNA versus GIT1-shRNA2, *P* < 0.0001; mRFP versus GIT1-mRFP, *P* = 0.0020; *t* tests) or without (**f**; *n* = 6, Ctrl versus DAPT, *P* < 0.0001; LV-Ctrl-shRNA versus LV-GIT1-shRNA4, *P* = 0.015; LV-mRFP versus LV-GIT1-mRFP, *P* = 0.0015; *t* tests) immobilized Jagged1 and as indicated. All data are shown as the mean ± s.e.m. *n* denotes the number of biologically independent replicates. **P* < 0.05, ***P* < 0.01, *****P* < 0.0001, ns, not significant by two-sided unpaired *t* tests. Source data are provided as a Source Data file.

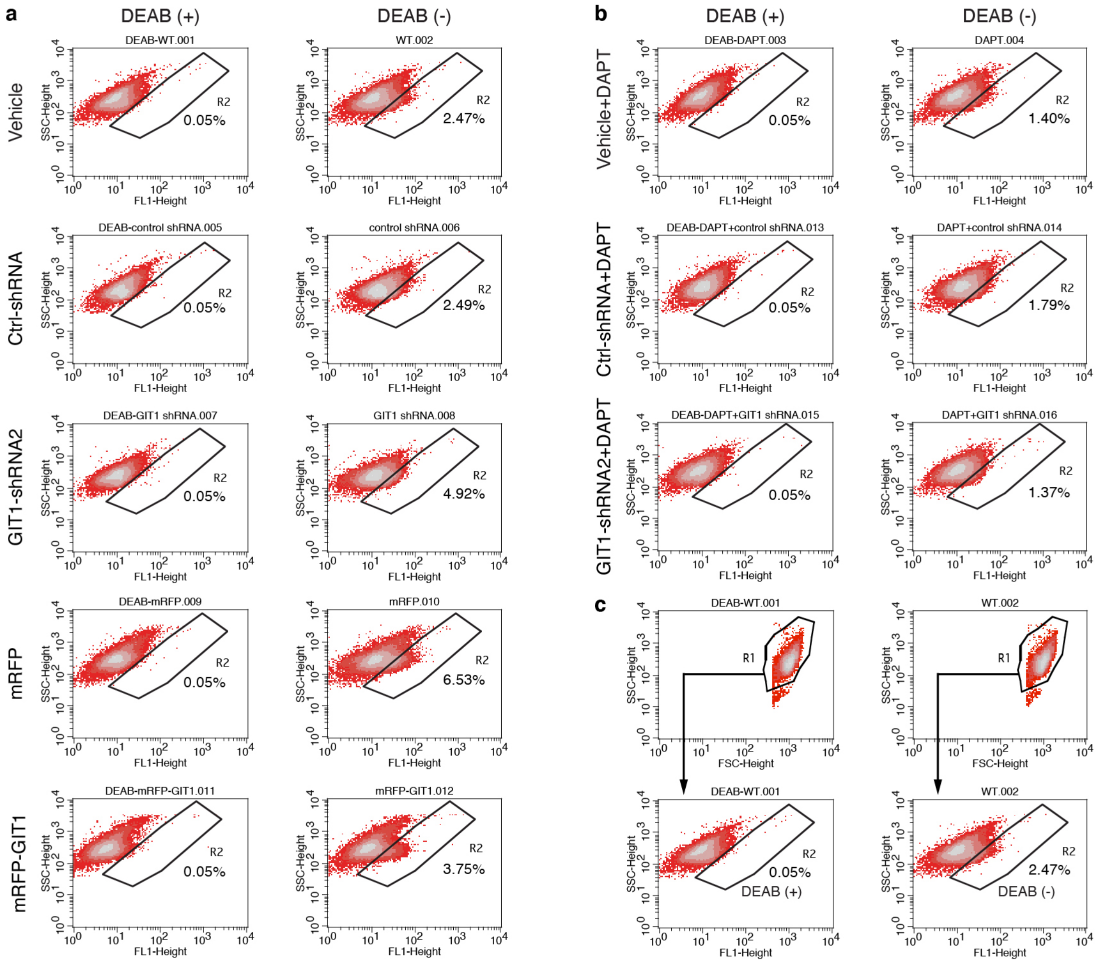


**Supplementary Figure 4 | Flow cytometric analysis of ALDH in breast cancer cells. a, b,** Flow cytometric analysis of Aldefluor assayed MDA-MB-231 cells treated as indicated without (**a**) or with (**b**) 50 µM DAPT. Control with 15 mM diethylaminobenzaldehyde (DEAB), a specific ALDH inhibitor. **c**, Gating strategy for flow cytometry analysis of Aldefluor-stained samples. First, we gated on ALDH positive cells (R1) by using forward versus side scatter (upper panels) and then we gated on DEAB inhibition (R2) by using fluorescence light versus side scatter (lower panels). SSC-Height, Side scatter light; FSC-Height, Forward scatter light; and FL1-Height, ALDEFLUOR light.

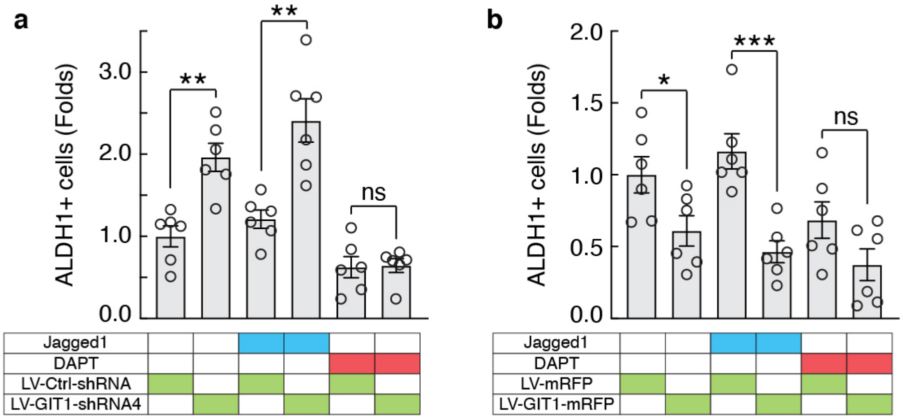


**Supplementary Figure 5 | Activation of the GIT1-Notch axis increases ALDH1 activity in breast cancer cells.** **a**-**b**, Flow cytometric analysis of ALDH enzyme activity in Aldefluor-treated HCC1395 cells transduced with LV-GIT1-shRNA4 (**a**; $n$ = 6, LV-Ctrl-shRNA versus LV-GIT1-shRNA4, $P$ = 0.0011; LV-Ctrl-shRNA+Jagged1 versus LV-GIT1-shRNA4+Jagged1, $P$ = 0.0019; LV-Ctrl-shRNA+DAPT versus LV-GIT1-shRNA4+DAPT, $P$ = 0.91; $t$ tests) or LV-GIT1-mRFP (**b**; $n$ = 6, LV-mRFP versus LV-GIT1-mRFP, $P$ = 0.039; LV-mRFP+Jagged1 versus LV-GIT1-mRFP+Jagged1, $P$ = 0.0007; LV-mRFP+DAPT versus LV-GIT1-mRFP+DAPT, $P$ = 0.094; $t$ tests) treated as indicated. All data are shown as the mean ± s.e.m. $n$ denotes the number of biologically independent replicates. *$P < 0.05$, **$P < 0.01$, ***$P < 0.001$, ns, not significant by two-sided unpaired $t$ tests. Source data are provided as a Source Data file.

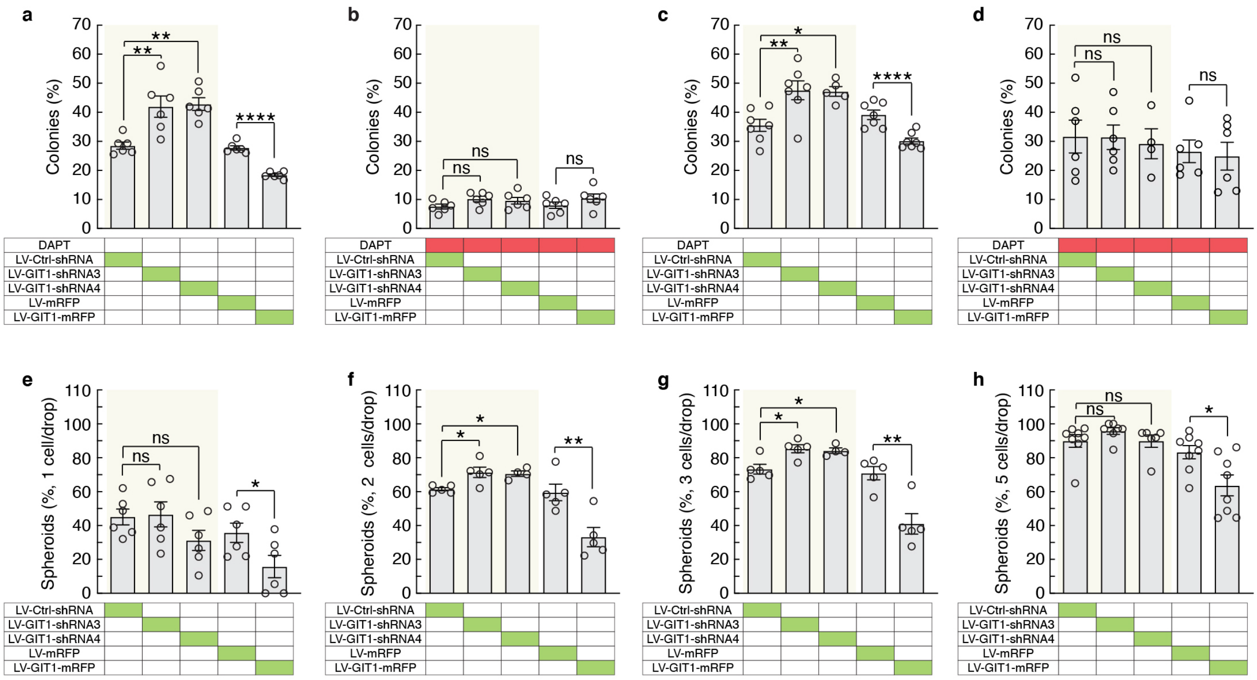


**Supplementary Figure 6 | Activation of the GIT1-Notch axis increases the colonization of breast cancer cells. a**-**b**, Clonogenic assays of MDA-MB-231 cells treated without (**a**; *n* = 6, LV-Ctrl versus LV-GIT1-shRNA3, *P* = 0.0056; LV-Ctrl versus LV-GIT1-shRNA4, *P* = 0.0035; one-way ANOVA, $F_{2,15}$ = 9.787, *P* = 0.0019 (shaded area); LV-mRFP versus LV-GIT1-mRFP, *P* < 0.0001; *t* tests) or with DAPT (**b**; *n* = 6, LV-Ctrl versus LV-GIT1-shRNA3, *P* = 0.18; LV-Ctrl versus LV-GIT1-shRNA4, *P* = 0.35; one-way ANOVA, $F_{2,15}$ = 1.915, *P* = 0.18 (shaded area); LV-mRFP versus LV-GIT1-mRFP, *P* = 0.42; *t* tests) and as indicated. **c**-**d**, Clonogenic assays of HCC1395 cells treated without (**c**; LV-Ctrl (*n* = 7) versus LV-GIT1-shRNA3 (*n* = 7), *P* = 0.0088; LV-Ctrl (*n* = 7) versus LV-GIT1-shRNA4 (*n* = 5), *P* = 0.020; one-way ANOVA, $F_{2,16}$ = 7.314, *P* = 0.0055 (shaded area); LV-mRFP (*n* = 7) versus LV-GIT1-mRFP (*n* = 7), *P* = 0.0005; *t* tests) or with DAPT (**d**; LV-Ctrl (*n* = 6) versus LV-GIT1-shRNA3 (*n* = 6), *P* = 0.99; LV-Ctrl (*n* = 6) versus LV-GIT1-shRNA4 (*n* = 4), *P* = 0.94; one-way ANOVA, $F_{2,13}$ = 0.05881, *P* = 0.94 (shaded area); LV-mRFP (*n* = 6) versus LV-GIT1-mRFP (*n* = 6), *P* = 0.80; *t* tests) and as indicated. **e**-**h**, Hanging-drop spheroid assays of HCC1395 cells seeded at 1 cell/drop (**e**; *n* = 6, LV-Ctrl versus LV-GIT1-shRNA3, *P* = 0.98; LV-Ctrl versus LV-GIT1-shRNA4, *P* = 0.27; one-way ANOVA, $F_{2,15}$ = 1.934, *P* = 0.18 (shaded area); LV-mRFP versus LV-GIT1-mRFP, *P* = 0.046; *t* tests), 2 cells/drop (**f**; LV-Ctrl (*n* = 5) versus LV-GIT1-shRNA3 (*n* = 5), *P* = 0.014; LV-Ctrl (*n* = 5) versus LV-GIT1-shRNA4 (*n* = 4), *P* = 0.031; one-way ANOVA, $F_{2,11}$ = 7.098, *P* = 0.011 (shaded area); LV-mRFP (*n* = 5) versus LV-GIT1-mRFP (*n* = 5), *P* = 0.0080; *t* tests), 3 cells/drop (**g**; LV-Ctrl (*n* = 5) versus LV-GIT1-shRNA3(*n* = 5), *P* = 0.010; LV-Ctrl (*n* = 5) versus LV-GIT1-shRNA4 (*n* = 4), *P* = 0.026; one-way ANOVA, $F_{2,11}$ = 7.747, *P* = 0.0080 (shaded area); LV-mRFP (*n* = 5) versus LV-GIT1-mRFP (*n* = 5), *P* = 0.0032; *t* tests), or 5 cells/drop (**h**; LV-Ctrl (*n* = 8) versus LV-GIT1-shRNA3 (*n* = 7), *P* = 0.40; LV-Ctrl (*n* = 8) versus LV-GIT1-shRNA4 (*n* = 6), *P* = 1.00; one-way ANOVA, $F_{2,18}$ = 1.105, *P* = 0.35 (shaded area); LV-mRFP (*n* = 8) versus LV-GIT1-mRFP (*n* = 8), *P* = 0.018; *t* tests) and treated as indicated. All data are shown as the mean ± s.e.m. *n* denotes the number of biologically independent replicates. **P* < 0.05, ***P* < 0.01, *****P* < 0.0001, ns, not significant by two-sided unpaired *t* tests or one-way ANOVA with Tukey's post hoc comparison. Source data are provided as a Source Data file.

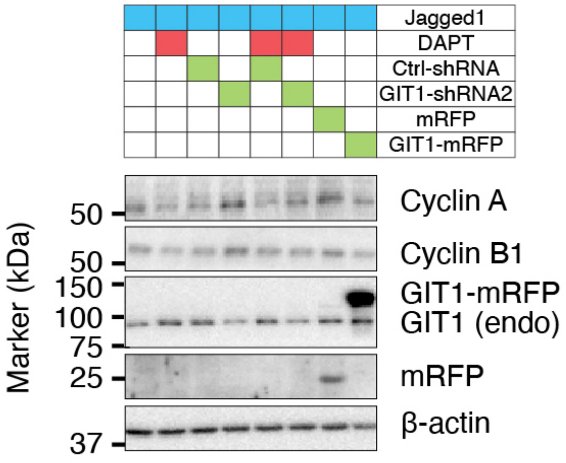


**Supplementary Figure 7 | The GIT1-Notch axis controls cyclin A and B1 in breast cancer cells.** Western blot of Cyclin A and Cyclin B1 in MDA-MB-231 cells treated as indicated. Images from representative blots; the experiment was repeated *n* = 3 times with similar results. Source data are provided as a Source Data file.

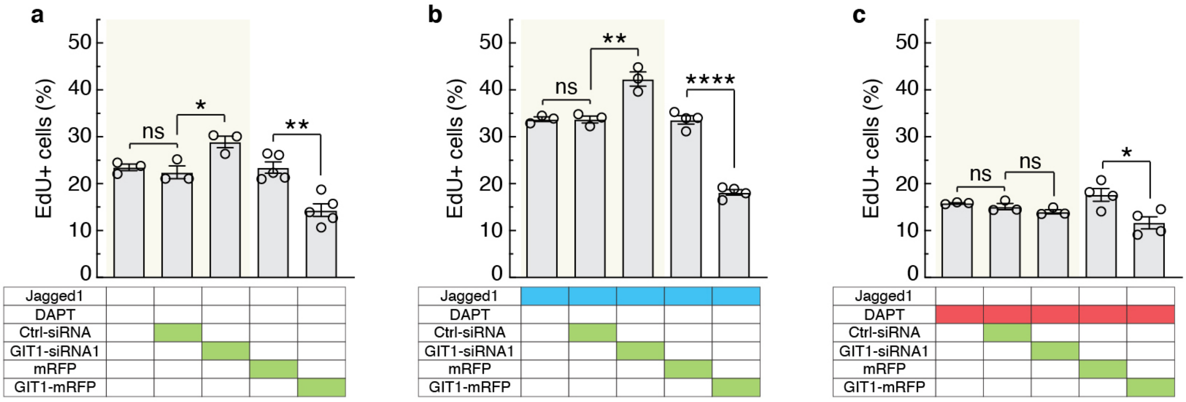


**Supplementary Figure 8 | Activation of the GIT1-Notch axis stimulates growth of breast cancer cells. a-c**, Percentage of EdU-positive (EdU+) MDA-MB-231 cells treated without Jagged1 (**a**; *n* = 3, Ctrl versus Ctrl-siRNA, *P* = 0.80; Ctrl-siRNA versus GIT1-siRNA1, *P* = 0.036; one-way ANOVA, $F_{2,6}$ = 9.160, *P* = 0.015 (shaded area); *n* = 5, mRFP versus GIT1-mRFP, *P* = 0.0010, *t* test), with Jagged1 (**b**; *n* = 3, Ctrl versus Ctrl-siRNA, *P* = 1.00; Ctrl-siRNA versus GIT1-siRNA1, *P* = 0.0024; one-way ANOVA, $F_{2,6}$ = 23.81, *P* = 0.0014 (shaded area); *n* = 4, mRFP versus GIT1-mRFP, *P* < 0.0001, *t* test), or with DAPT (**c**; *n* = 3, Ctrl versus Ctrl-siRNA, *P* = 0.55; Ctrl-siRNA versus GIT1-siRNA1, *P* = 0.073; one-way ANOVA, $F_{2,6}$ = 3.883, *P* = 0.083 (shaded area); *n* = 4, mRFP versus GIT1-mRFP, *P* = 0.020, *t* test) and as indicated. All data are shown as the mean ± s.e.m. *n* denotes the number of biologically independent replicates. **P* < 0.05, ***P* < 0.01, ****P* < 0.001, *****P* < 0.0001, ns, not significant by two-sided unpaired *t* tests or one-way ANOVA with Tukey's post hoc comparison. Source data are provided as a Source Data file.

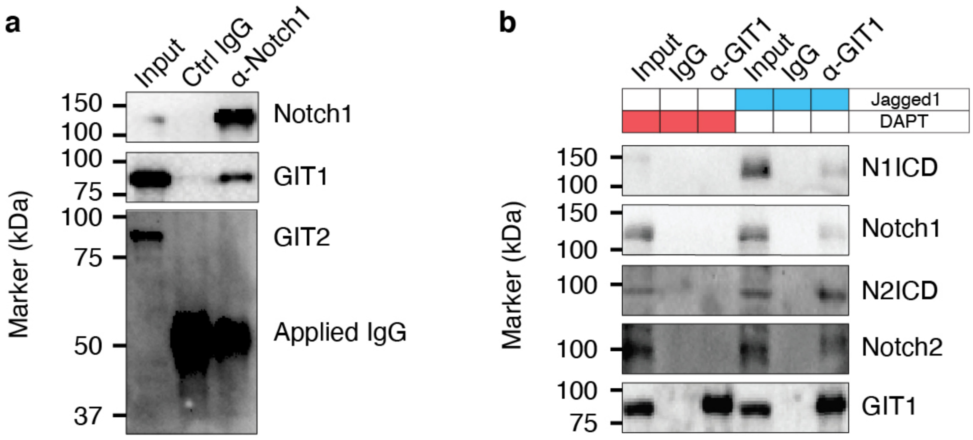


**Supplementary Figure 9 | GIT1, but not GIT2, binds to Notch1 and Notch2. a**, Coimmunoprecipitation of Notch1 with GIT1 and GIT2 from the lysates of 184A1 cells. **b**, Coimmunoprecipitation of Notch1, Notch2, and their ICDs with GIT1 from the lysates of HCC1395 cells treated as indicated. Images from representative blots; the experiment was repeated *n* = 3 times with similar results. Source data are provided as a Source Data file.

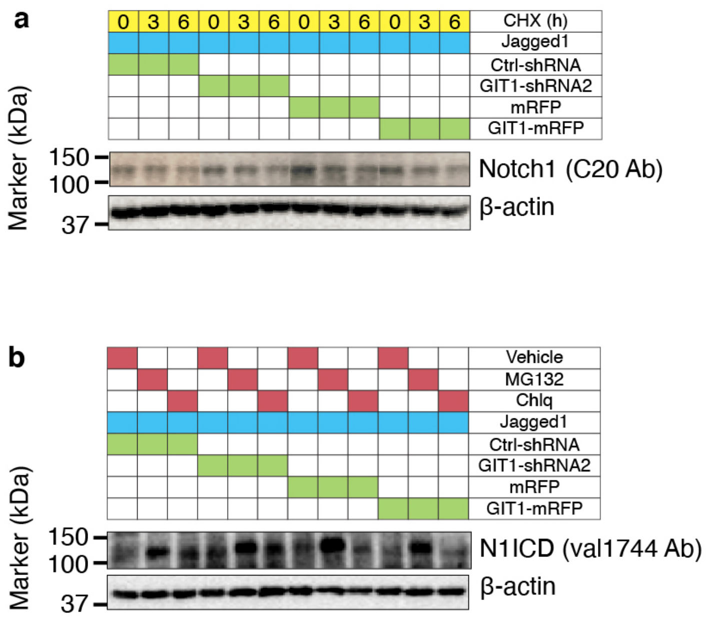


**Supplementary Figure 10 | GIT1 does not alter Notch synthesis and degradation. a**, **b**, Western blots of MDA-MB-231 cells treated with 20 µg/ml cycloheximide (CHX) for 0, 3, or 6 h (**a**), or with 40 µM MG132 or 40 µM Chloroquine (Chlq) for 24 h (**b**). Cycloheximide (CHX) is inhibiting protein biosynthesis, MG132 is inhibiting proteasomal degradation, and Chloroquine (Chlq) is inhibiting lysosomal degradation. Images from representative blots; the experiment was repeated *n* = 2 times with similar results. Source data are provided as a Source Data file.

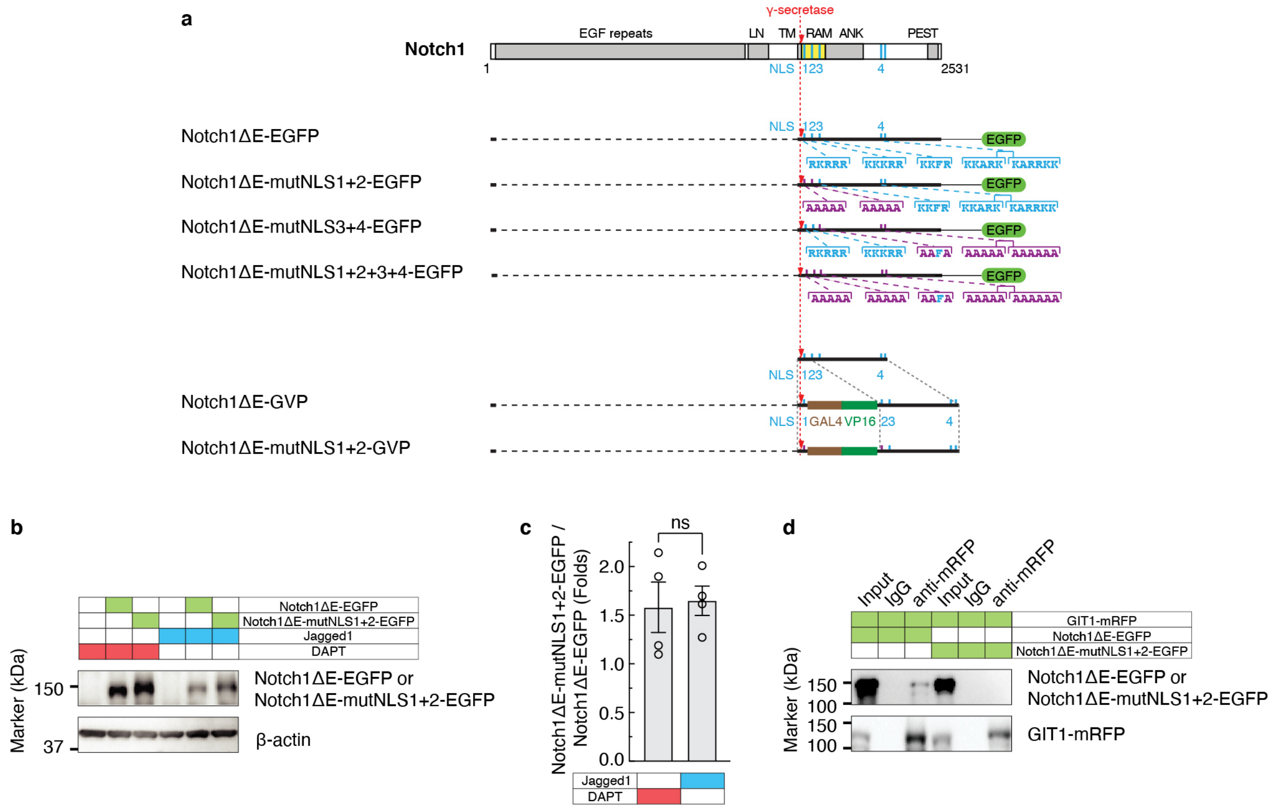


**Supplementary Figure 11 | GIT1 binds to Notch NLS1 and NLS2. a**, Schematics of the used plasmids. **b**-**c**, Western blots (**b**) and the quantitative immunoblot analysis of the EGFP intensity ratio between the lysates of MDA-MB-231 cells expressing Notch1ΔE-mutNLS1+2-EGFP or Notch1ΔE-EGFP (**c**, $n$ = 4, $P$ = 0.83, $t$ tests) treated with DAPT or Jagged1. **d**, Coimmunoprecipitation of GIT1-mRFP with Notch1ΔE-EGFP or Notch1ΔE-mutNLS1+2-EGFP from the lysates of transfected MDA-MB-231 cells. Image from representative blot; the experiment was repeated $n$ = 2 times with similar results. All data are shown as the mean ± s.e.m. $n$ denotes the number of biologically independent replicates. ns, not significant by two-sided unpaired $t$ tests. Source data are provided as a Source Data file.

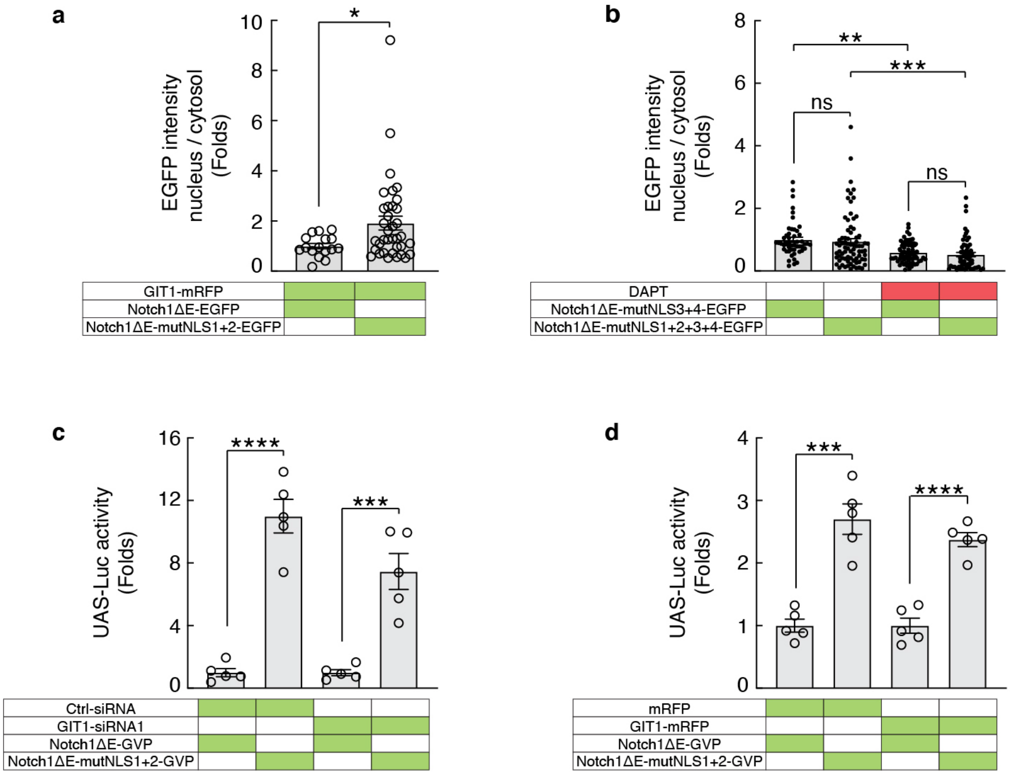


**Supplementary Figure 12 | GIT1 regulates Notch nuclear translocation via NLS1 and NLS2. a, b**, Quantitative immunocytochemistry analysis of the EGFP intensity ratio between the cytoplasm and nucleus in MDA-MB-231 cells expressing GIT1-mRFP plus Notch1ΔE-EGFP or Notch1ΔE-mutNLS1+2-EGFP (**a**; GIT1-mRFP+Notch1ΔE-EGFP (*n* = 16 cells) versus GIT1-mRFP+Notch1ΔE-mutNLS1+2-EGFP (*n* = 36 cells), *P* = 0.037, *t* test) and Notch1ΔE-mutNLS3+4-EGFP or Notch1ΔE-mutNLS1+2+3+4-EGFP with or without DAPT (**b**; Notch1ΔE-mutNLS3+4-EGFP (*n* = 49 cells) versus Notch1ΔE-mutNLS1+2+3+4-EGFP (*n* = 77 cells), *P* = 0.95; Notch1ΔE-mutNLS3+4-EGFP versus Notch1ΔE-mutNLS3+4-EGFP+DAPT (*n* = 71 cells), *P* = 0.0019; Notch1ΔE-mutNLS3+4-EGFP+DAPT versus Notch1ΔE-mutNLS1+2+3+4-EGFP+DAPT, *P* = 0.92; Notch1ΔE-mutNLS1+2+3+4-EGFP versus Notch1ΔE-mutNLS1+2+3+4-EGFP+DAPT (*n* = 60 cells), *P* = 0.0005; one-way ANOVA, $F_{3,253}$ = 9.742, *P* < 0.0001). **c, d**, Luciferase reporter assays of Notch1 ICD (UAS-Luc) in MDA-MB-231 cells with knockdown (**c**; *n* = 5 independent biological replicates, Ctrl-siRNA+Notch1ΔE-GVP versus Ctrl-siRNA+Notch1ΔE-mutNLS1+2-GVP, *P* < 0.0001; GIT1-siRNA1+Notch1ΔE-GVP versus GIT1-siRNA1+Notch1ΔE-mutNLS1+2-GVP, *P* = 0.0005; *t* tests) or overexpression (**d**; *n* = 5 independent biological replicates, mRFP+Notch1ΔE-GVP versus mRFP+Notch1ΔE-mutNLS1+2-GVP, *P* = 0.0002; GIT1-mRFP+Notch1ΔE-GVP versus GIT1-mRFP+Notch1ΔE-mutNLS1+2-GVP, *P* < 0.0001; *t* tests) of GIT1, treated as indicated. All data are shown as the mean ± s.e.m. ns, not significant, **P* < 0.05, ***P* < 0.01, ****P* < 0.001, *****P* < 0.0001 by two-sided unpaired *t* tests or one-way ANOVA with Tukey's post hoc comparison. Source data are provided as a Source Data file.

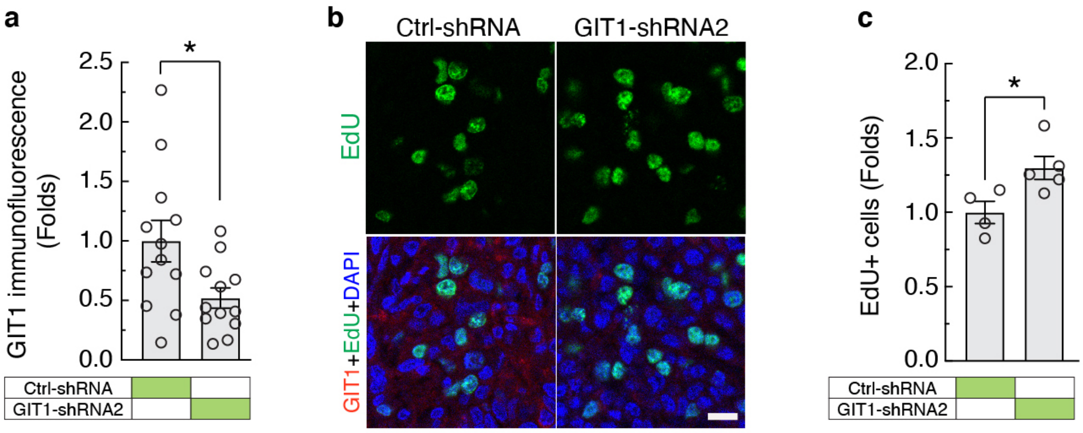


**Supplementary Figure 13 | GIT1 knockdown increases EdU staining in breast cancer xenografts**. **a**-**c**, Quantitative immunofluorescence analysis of GIT1 (**a**; *n* = 12 tumours, *P* = 0.022, *t* test), EdU staining (**b**) and the quantitative analysis (**c**; Ctrl-shRNA (*n* = 4 tumours) versus GIT1-shRNA2 (*n* = 5 tumours), *P* = 0.029, *t* test) in tumours from mice xenografted with MDA-MB-231 cells. Scale bar, 10 μm. All data are shown as the mean ± s.e.m. ns, not significant, **P* < 0.05 by two-sided unpaired *t* tests. Source data are provided as a Source Data file.

# Supplementary Tables

| Accession codes | Hyperlinks |
|---|---|
| E-MTAB-365 | [https://www.ebi.ac.uk/arrayexpress/experiments/E-MTAB-365/] |
| GSE11121 | [https://www.ncbi.nlm.nih.gov/geo/query/acc.cgi?acc=GSE11121] |
| GSE12093 | [https://www.ncbi.nlm.nih.gov/geo/query/acc.cgi?acc=GSE12093] |
| GSE12276 | [https://www.ncbi.nlm.nih.gov/geo/query/acc.cgi?acc=GSE12276] |
| GSE1456 | [https://www.ncbi.nlm.nih.gov/geo/query/acc.cgi?acc=GSE1456] |
| GSE16391 | [https://www.ncbi.nlm.nih.gov/geo/query/acc.cgi?acc=GSE16391] |
| GSE16446 | [https://www.ncbi.nlm.nih.gov/geo/query/acc.cgi?acc=GSE16446] |
| GSE16716 | [https://www.ncbi.nlm.nih.gov/geo/query/acc.cgi?acc=GSE16716] |
| GSE17705 | [https://www.ncbi.nlm.nih.gov/geo/query/acc.cgi?acc=GSE17705] |
| GSE17907 | [https://www.ncbi.nlm.nih.gov/geo/query/acc.cgi?acc=GSE17907] |
| GSE19615 | [https://www.ncbi.nlm.nih.gov/geo/query/acc.cgi?acc=GSE19615] |
| GSE20271 | [https://www.ncbi.nlm.nih.gov/geo/query/acc.cgi?acc=GSE20271] |
| GSE2034 | [https://www.ncbi.nlm.nih.gov/geo/query/acc.cgi?acc=GSE2034] |
| GSE20685 | [https://www.ncbi.nlm.nih.gov/geo/query/acc.cgi?acc=GSE20685] |
| GSE20711 | [https://www.ncbi.nlm.nih.gov/geo/query/acc.cgi?acc=GSE20711] |
| GSE21653 | [https://www.ncbi.nlm.nih.gov/geo/query/acc.cgi?acc=GSE21653] |
| GSE25066 | [https://www.ncbi.nlm.nih.gov/geo/query/acc.cgi?acc=GSE25066] |
| GSE2603 | [https://www.ncbi.nlm.nih.gov/geo/query/acc.cgi?acc=GSE2603] |
| GSE26971 | [https://www.ncbi.nlm.nih.gov/geo/query/acc.cgi?acc=GSE26971] |
| GSE2990 | [https://www.ncbi.nlm.nih.gov/geo/query/acc.cgi?acc=GSE2990] |
| GSE31519 | [https://www.ncbi.nlm.nih.gov/geo/query/acc.cgi?acc=GSE31519] |
| GSE3494 | [https://www.ncbi.nlm.nih.gov/geo/query/acc.cgi?acc=GSE3494] |
| GSE37946 | [https://www.ncbi.nlm.nih.gov/geo/query/acc.cgi?acc=GSE37946] |
| GSE42568 | [https://www.ncbi.nlm.nih.gov/geo/query/acc.cgi?acc=GSE42568] |
| GSE45255 | [https://www.ncbi.nlm.nih.gov/geo/query/acc.cgi?acc=GSE45255] |
| GSE4611 | [https://www.ncbi.nlm.nih.gov/geo/query/acc.cgi?acc=GSE4611] |
| GSE46184 | [https://www.ncbi.nlm.nih.gov/geo/query/acc.cgi?acc=GSE46184] |
| GSE48390 | [https://www.ncbi.nlm.nih.gov/geo/query/acc.cgi?acc=GSE48390] |
| GSE4922 | [https://www.ncbi.nlm.nih.gov/geo/query/acc.cgi?acc=GSE4922] |
| GSE5327 | [https://www.ncbi.nlm.nih.gov/geo/query/acc.cgi?acc=GSE5327] |
| GSE61304 | [https://www.ncbi.nlm.nih.gov/geo/query/acc.cgi?acc=GSE61304] |
| GSE65194 | [https://www.ncbi.nlm.nih.gov/geo/query/acc.cgi?acc=GSE65194] |
| GSE6532 | [https://www.ncbi.nlm.nih.gov/geo/query/acc.cgi?acc=GSE6532] |
| GSE69031 | [https://www.ncbi.nlm.nih.gov/geo/query/acc.cgi?acc=GSE69031] |
| GSE7390 | [https://www.ncbi.nlm.nih.gov/geo/query/acc.cgi?acc=GSE7390] |
| GSE9195 | [https://www.ncbi.nlm.nih.gov/geo/query/acc.cgi?acc=GSE9195] |

**Supplementary Table 1 | Accession codes for publicly available breast cancer data sets.** Accession codes for publicly available breast cancer data sets that were analysed with KM-plotter.
